# Extreme Optical Chirality from Plasmonic Nanocrystals on a Mirror

**DOI:** 10.1021/acs.nanolett.4c05668

**Published:** 2025-01-13

**Authors:** Yidong Hou, Xiu Yang, Shu Hu, Qianqi Lin, Jie Zhou, Jialong Peng, Chenyang Guo, Shanshan Huang, Liangke Ren, Ana Sánchez-Iglesias, Rohit Chikkaraddy, Jeremy J. Baumberg

**Affiliations:** † College of Physical Science and Technology, Sichuan University, China, Chengdu 610065, China; ‡ NanoPhotonics Centre, Cavendish Laboratory, Department of Physics, University of Cambridge, Cambridge CB3 0HE, United Kingdom; § CIC biomaGUNE, Basque Research and Technology Alliance (BRTA), Donostia-San Sebastián 20014, Spain; ∥ Center of Materials Physics, CSIC-UPV, Donostia-San Sebastián 20018, Spain; ⊥ School of Physics and Astronomy, University of Birmingham, Birmingham B15 2TT, United Kingdom; ∇ College of Advanced Interdisciplinary Studies and Hunan Provincial Key Laboratory of Novel Nano-Optoelectronic Information Materials and Devices, National University of Defense Technology, Changsha 410073, China

**Keywords:** nanodecahedra, chiroptical effect, nanoparticle on mirror, multiple dipole decomposition theory, photonic spin Hall effect

## Abstract

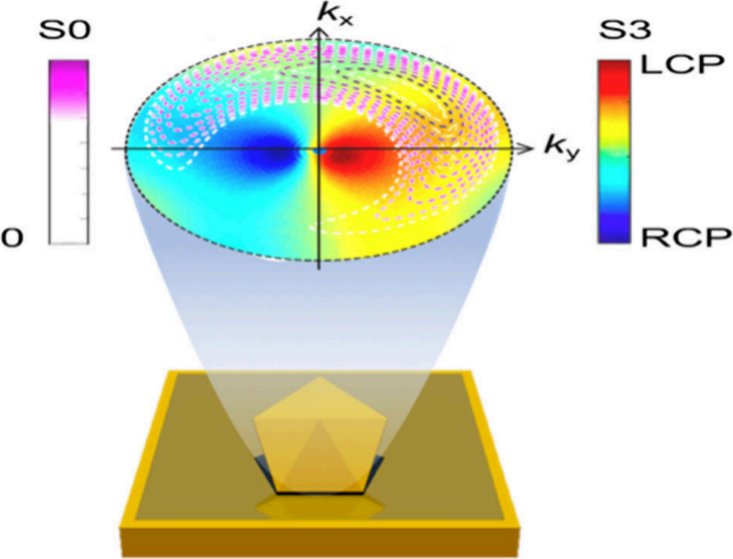

Metal nanocrystals synthesized in achiral environments usually exhibit no chiroptical effects. However, by placing nominally achiral nanocrystals 1.3 nm above gold films, we find giant chiroptical effects, reaching anisotropy factors as high as *g* ≈ 0.9 for single nanodecahedra placed on a gold mirror (NDoM). We show that this is a general phenomenon depending on the geometry, demonstrating it for various nanocrystal shapes. Theoretical modeling reveals that tiny chiral imperfections are strongly enhanced by edge modes in the gap, which coherently superpose with in-plane dipoles to generate strong chiroptical signatures. This phenomenon results in photonic spin Hall effects and distinctive chiral scattering patterns.

Chirality describes a geometry or point group without mirror symmetry: e.g., a helix. Interaction of chiral geometries with light induces cross-coupling between electric and magnetic dipoles in a medium and splits the response for left and right circularly polarized (LCP and RCP) optical illumination. These chiroptical effects pave the way for recognizing stereochemical structures of molecules and to manipulate the polarization state of light in photonic applications.^1−3^ The strongest effects so far are exhibited in chiral metamaterials, which show unusual physical behavior including optical activity in diffraction,^4^ negative refractive index,^5−8^ optical spin Hall effects,^9,10^ and applications such as chiral biosensors.^11−13^

Synthesizing materials with chiral geometry is difficult. Often top-down methods such as optical and e-beam lithography have been used, but they suffer from limited scalability and involve complex technological processes.^14−18^ Bottom-up methods^19−22^ such as wet-chemical synthesis require precise control of the microscopic self-assembly process that so far remains challenging. Creation of chiral metal nanocrystals has been recently improved by the direct synthesis of chiral plasmonic helicoids.^23^ However, most plasmonic nanocrystals typically feature either symmetric or random morphologies and are therefore achiral. As a result, chiral metamaterials have been typically realized by assembling achiral plasmonic nanocrystals onto chiral molecular templates^3,22,24^ such as polymer fibers,^25,26^ peptides,^27,28^ and DNA^29,30^ (Table S1).

Here, by placing nominally achiral plasmonic nanocrystals just a few nanometers above a metal film (mirror), we present both experimental and theoretical demonstrations that a giant chiroptical effect arises in light scattering. Reversing normal assumptions,^20,23,31−35^ we find that almost all plasmonic nanocrystals are slightly irregular and possess either a chiral shape or lattice imperfections that can induce chiroptical effects in scattering.^36^ This spontaneously formed chirality effect is often extremely weak and random for each crystal, and thus the ensemble of many nanocrystals is racemic, leading to extremely weak chiroptical effects that mask any spontaneously formed chirality.^37,38^

To observe this spontaneously formed chirality, we construct a single-particle polarization scattering measurement system (Figure 1a) to compare with recent measurements (Table S1).^32,39−43^ White light from a 100 W halogen source illuminates samples through a 100× dark-field objective, which collects the scattered light and divides it into LCP and RCP channels using a broad-band quarter-waveplate and polarization beamsplitter. The LCP and RCP scattering spectra and spatial images are measured on a charge-coupled device (CCD) camera coupled to a spectrometer. Overall scattering images are also recorded by temporarily inserting a beam splitter and CCD camera (red dashed outline). As an initial example, gold decahedral nanocrystals (NDs) are investigated due to their regular shape, which simplifies theory and numerical simulations. Images (Figure 1a insets) show a scanning electron microscopy (SEM) image of a ND and overall scattering patterns filtered around 700 nm for two individual nanodecahedra-on-mirror (NDoMs), divided into LCP and RCP images where the ND is spaced 1.3 nm above the mirror by an inert single molecular spacer of biphenyl-4-thiol (BPT, see SI Methods). Crescent-shaped scattering patterns are seen, with significant differences between LCP and RCP. Their shape originates from antenna dipoles formed in the ND, which are tilted from the substrate normal, while the difference between LCP and RCP comes from strong spin–orbit interactions, as will be shown by the detailed analysis discussed below.

**Figure 1:**
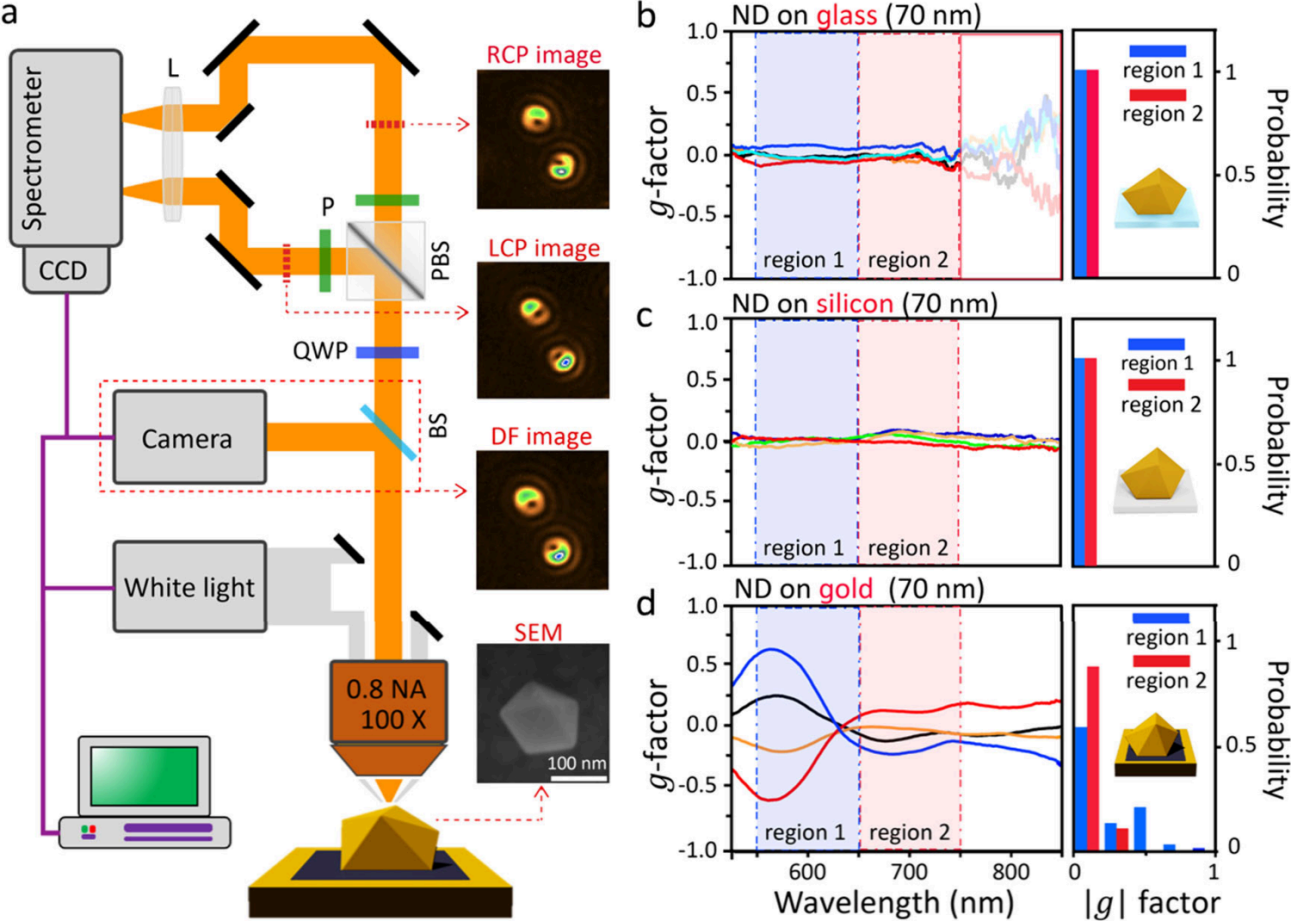
(a) Schematic of polarization-dependent darkfield scattering. Inset images (right panels) are SEM and scattering patterns of NDoMs. Definitions: lens (L), quarter-waveplate (QWP), beam splitter (BS), polarization beam splitter (PBS), and polarizer (P). (b–d) Measured scattering *g*-factor spectra of individual nanodecahedra on different substrates made of (b) glass, (c) silicon, and (d) BPT-coated gold. The right panel shows the frequency of finding the maximum *g*-factor in spectral regions 1 (blue) and 2 (red).

The NDoM LCP and RCP scattering spectra from spatial integration of the scattering patterns yield the corresponding *g*-factor spectra, through the following equation: *g*(λ) = 2(RCP – LCP)/(RCP + LCP) (Figure 1b–d). For NDs placed on glass (Figure 1b) or silicon (Figure 1c), the scattering *g*-factor from NDs is <0.1 and is inconsistent across all spectral ranges (note the poor signal-to-noise ratio for λ > 750 nm, due to weak scattering signals). For NDs spaced ∼1 nm above gold, remarkably intense *g*-factors are obtained, especially within the range 550 nm < λ < 650 nm (region 1), where it can even reach ∼0.9. This value exceeds all values reported for chiral nanocrystals (Table S1),^32,39−43^ and can be further increased by choosing specific illumination conditions (Figures S2 and S3). This giant chiroptical effect indicates that spontaneously formed chirality can be comparable with complex, intentionally chiral structures, and thus NDoMs may find wide application in nanophotonics. The slightly low symmetry or imperfect morphology of each ND is however random, leading to distinct *g*(λ)-factor spectra for different NDs, with some NDs showing positive peaks in *g*(λ) and others negative. In complete contrast to NDs on glass or Si, more than 40% of NDoMs possess maximum *g*-factors >0.1 in region 1, with an average of 0.3. This consistently strong chiroptical effect suggests a significant enhancement due to coupling to the gold film.

To reveal the origin of chiroptical effects, we investigate the geometry of NDs in TEM (Figure 2a).^44^ Each image reveals the different angular facet widths around the central vertex of NDs, which sets the ND chirality.^45^ This spontaneously formed chirality is random, with both LH- and RH-NDoMs found in any sample batch, as shown by two typical *g*-factor spectra in Figure 2b. The weak chiroptical effect of single NDs and their racemic averaging make spontaneously formed chirality hidden from traditional detection methods.

**Figure 2:**
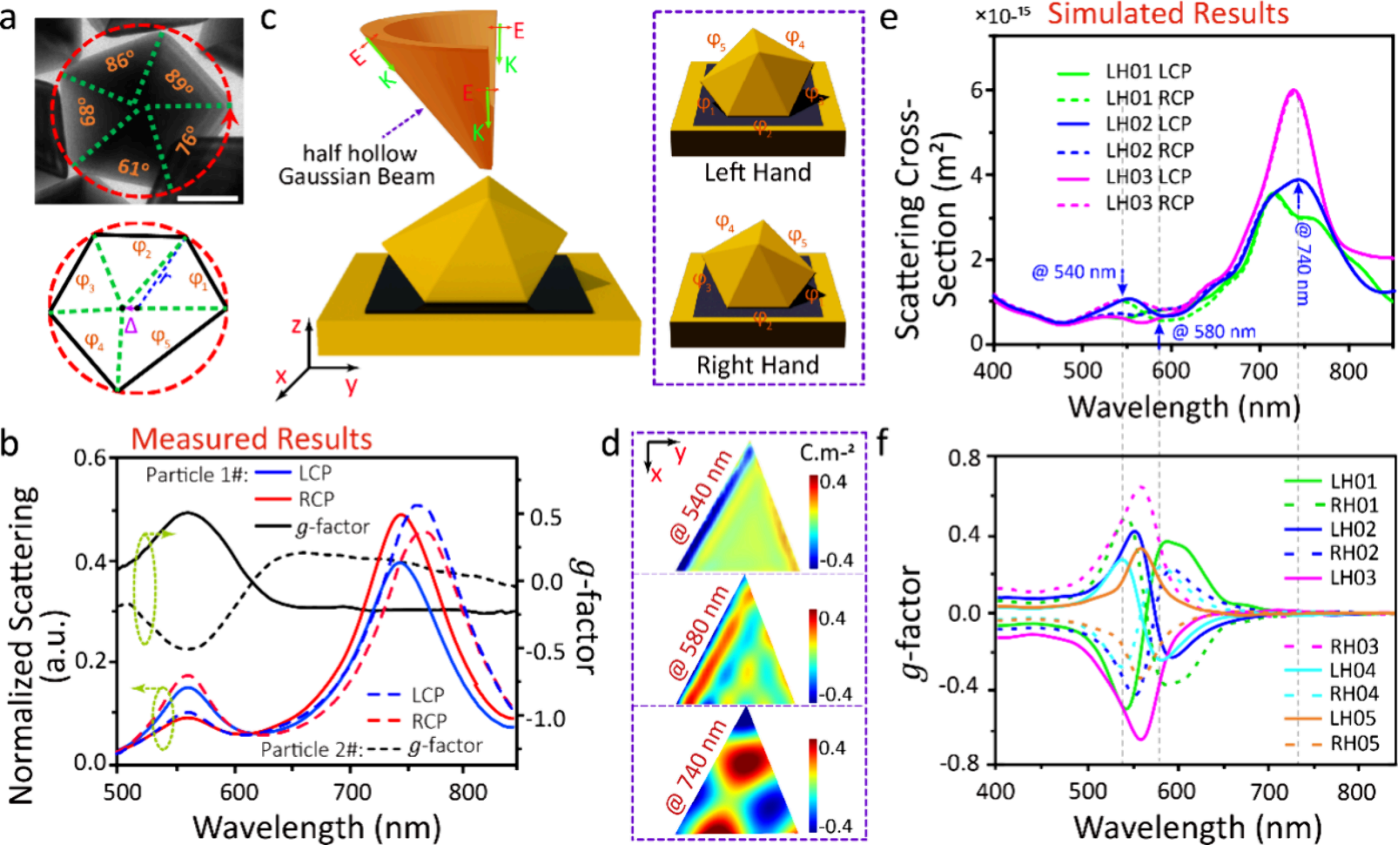
(a) Top-view TEM image of a typical ND (top) and central cross-section of ND model used in simulations (bottom). *r* denotes the radius of encompassing circle, Δ denotes the displacement between the ND vertex and circle center, and φ_*i*_ denote angles between facets (green dashed lines), labeled on the TEM image. The inset scale bar is 50 nm. (b) Measured LCP and RCP scattering spectra and *g*(λ) spectra of two typical NDoMs. (c) Schematic of half-hollow Gaussian beam illuminating NDoM (left) and the LH-/RH-NDoM models of structure #2 with five upper facets. (d) Simulated charge distributions on bottom surface facet of LH-NDoM model #2 shown in (c). (e) Simulated scattering cross-section and (f) *g*(λ) of the investigated NDoM models. Detailed geometrical and simulation information is given in Figures S4–S26.

To simulate this spontaneously formed chiral geometry, we start with ideal NDs formed by two pentagonal pyramids built from regular pentagons (Figure S4). The pentagonal edges are then adjusted to make spiralling facet-angles at the vertex, φ_1–5_ = 50, 61, 72, 83, and 94°. To further decrease the symmetry, the equatorial projection of the pyramidal apex is shifted slightly from the encircling center, by a distance Δ = 10 nm. The final geometrical model retains the pyramidal height (Figure S5, detailed parameters in Figure S4). The NDoM model is then created by placing this decahedron on a gold film with one facet facing down. Ten different possible lower facets create 10 NDoMs, in pairs of NDoM enantiomers each denoted by their edge number (Figure S6).

The NDoM optical properties are investigated using finite-difference time-domain methods (FDTD). To match the experimental illumination, a hollow Gaussian beam is generated (details in SI), which is truncated on the ±X or ±Y sides when employing X- or Y-polarized light (Figure 2c shows NDoM illuminated by Y-polarized half-hollow Gaussian beam from +Y side). Complete simulations of one NDoM model require four runs for X- and Y-polarized states on the X/Y sides, extracting the total LCP and RCP scattering signals from each. The gap size and refractive index in the gap between ND and gold film are set to 1.3 nm and *n* = 1.45, respectively (as previously determined from the molecular monolayer used,^46^ details of simulations in SI). Simulated results (Figure 2e–f and Figures S7–S18) show distinct scattering and *g*-factor spectra from different NDoMs under X/Y polarized illumination. In general, two main scattering peaks are located near 550 and 750 nm, with strong *g*-factor peaks around 550 nm for all NDoMs. These results coincide very well with the measured spectra (Figures 1 and 2b), implying that the observed chiroptical effect indeed comes from this chiral facet geometry of the NDs.

Enhancement from the gold mirror is the key factor in seeing chiroptical effects from NDs. The substrate-dependent chiroptical effects observed (Figure 1b–d) are confirmed by simulated results (Figures S19–S22), with the maximum simulated *g*-factor of NDoMs being ∼400% larger than for NDs on glass or silicon. The dominant enhancement is attributed to “edge modes” formed in the NDoM nanogap around 560 nm (Figure 2d). The edge modes are robust to different illumination conditions and disappear for λ > 650 nm (Figures S23 and S24). By contrast, edge modes are weak for NDs on glass or silicon, confirming the enhancement from image charges across the extremely narrow metal-dielectric-metal nanogap (Figures S25 and S26).

The far-field scattering images can be understood via multiple dipole decomposition theory.^47^ The scattering from NDs on different substrate materials is summarized by the combined emission of in-plane and out-of-plane dipoles whose strength varies with wavelength (Figure 3a and Figures S27–S29). The edge modes formed in the short waveband λ < 600 nm suppress the formation of out-of-plane dipoles and enhance the chiroptical effect of NDoMs where their out-of-plane electric dipole Pz is weak. At longer wavelengths, increasing Pz rotates the in-plane dipole, which becomes tilted across the nanogap (Figure 3b). We note the images observed in the experiment are limited to NA = 0.8 (set by the objective lens), resulting in an incomplete circular scattering pattern thus shaped as a crescent (Figure 1a).

**Figure 3:**
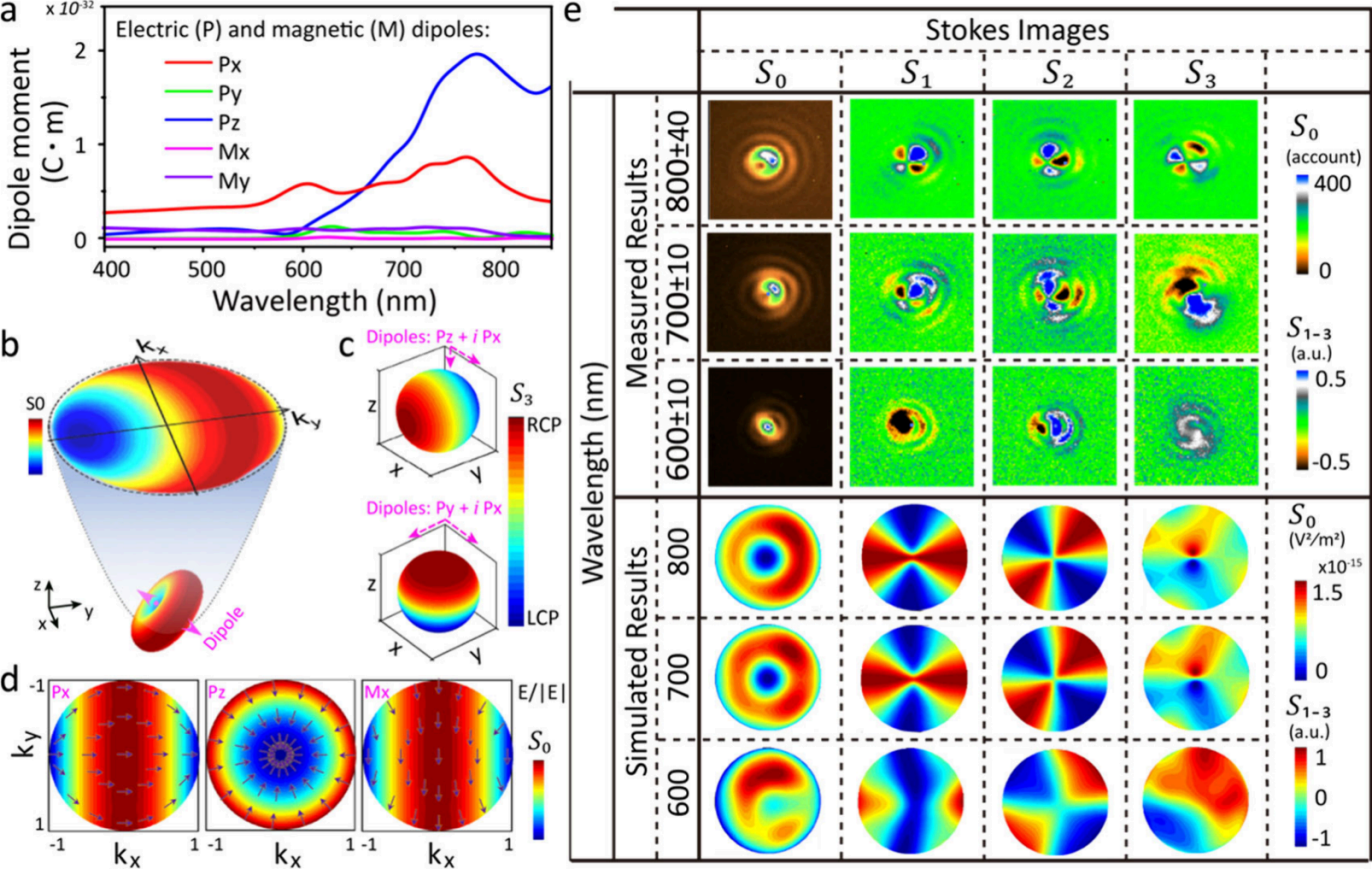
(a) Calculated dipole moments from LH NDoM model #2 under X-polarized illumination in the X- half space. (b) Schematic of collected scattering patterns from the back focal plane of a dipole tilted in the YZ plane. (c) Calculated *S*_3_ scattering patterns from ideal electric dipole of (top) Pz + *i*Px and (bottom) Py + *i*Px, both with Px phased 90° ahead. (d) Calculated scattering patterns and linear polarization states collected with an ideal lens from ideal electric dipoles along (left) X, (middle) Z, and (right) magnetic dipole along X. (e) Measured and simulated Stokes images at different spectral wavelengths of far-field scattering from an NDoM. The simulated NDoM model is LH #2, and simulated Stokes images sum those generated by half hollow Gaussian beams in X and Y polarizations. Detailed dipole emission and their superpositions are given in Figures S27–S35.

The strong out-of-plane dipole weakens the chiroptical effect in backscattering. Combining the dipoles Pz + *i*Px scatters RCP light toward −Y and LCP light toward +Y (Figure 3c). When collecting in the +Z direction, 50% is LCP-light and 50% is RCP-light, which leads to *g* = 0. This situation changes completely for combining in-plane dipoles Py + *i*Px, where RCP light scatters to +Z while LCP light scatters to −Z. In the +Z direction only RCP light is collected, leading to large *g* values. Oriented polarization-sensitive scattering is thus explained by coherent superposition of polarized emission from different dipoles (Figures S30–S32). Polarized dipole scattering possesses a symmetry axis along X for Px, along Y for Mx, and along Z for Pz (Figure 3d and Figure S31). Superposition of dipoles with the same symmetry axis in the polarization distributions of scattered light will not generate strong chiroptical effects (e.g., Px + Pz in Figure 3c), while the superposition of Px + Py breaks the scattering symmetry leading to strong chiroptical effects. (Figure S32) We note the out-of-plane magnetic dipole Mz and high-order dipoles are all ignored due to their near-zero intensity in this spectral region.

Full Stokes polarization scattering patterns (*S*_0–3_) further confirm the influence of out-of-plane dipoles. Both measured and simulated results (Figure 3e and Figures S33 and S34) show crescent-like *S*_0_ (intensity) patterns and *S*_3_ (chirality) patterns with near-equal negative and positive values for 700–800 nm, where the out-of-plane dipole Pz dominates the NDoM scattering. In addition, the four-leaf *S*_1,2_ scattering patterns come from the radially polarized scattered light of the out-of-plane dipole Pz (Figure 3d and Figure S35). Below 600 nm, the in-plane dipoles dominate NDoM scattering and the crescent-like *S*_0_ and four-leaf *S*_1,2_ patterns all deform. The coherent superposition between the in-plane dipoles scatters more RCP light to +Z, leading to a strong chiroptical effect in far-field. This implies that it is the in-plane dipoles that best sense the chiral geometry of NDs and give strong chiroptical effects. The *S*_3_ scattering patterns also indicate a strong chiral photonic spin Hall effect, where the LCP and RCP light scatters into different directions, with also distinct intensities.^10,48^ The microscopic mechanism behind such strong photonic spin Hall effects in nanocrystals placed on a mirror will be discussed elsewhere. For NDs placed on glass or silicon where no edge modes form, the calculated out-of-plane dipole Pz is never suppressed in these ranges (Figures S28 and S29), which leads to weak chiroptical effects.

The spontaneously formed chirality observed for NDs exists more widely in solution seed-growth of nanocrystals.^49,50^ Since these typically rely on organic ligands to select for faster growing facets, imbalances in growth rates on different sides will generally form random facet chirality, which accumulates over growth time. This may be either during the growth phase or inherited from the seed, although this chirality remains weak and uncontrollable in the achiral synthetic environment. Larger NDs give a stronger chiroptical effect when the facet edge increases from 40 to 80 nm (Figure 4a–c and Figure S36), increasing from 30% to 60%, while the chiroptical resonance peak also shifts to longer wavelengths along with the localized plasmonic resonances.^46^

**Figure 4:**
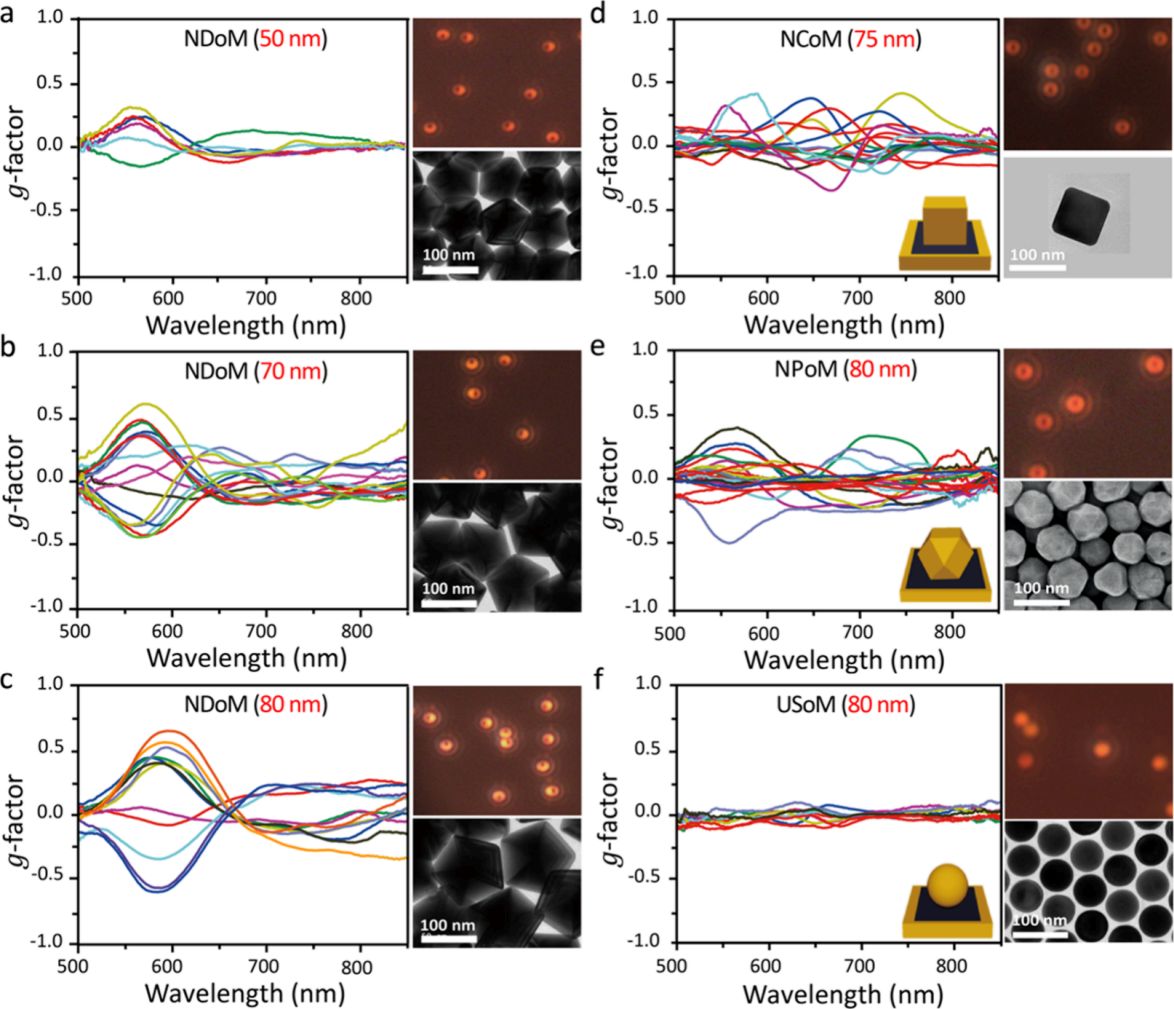
Measured *g*-factor spectra of many different shaped nanocrystals. (a–c) NDoMs with different sizes as labeled, (d) NCoM, (e) cuboctahedral NPoM, and (f) ultraspherical USoM. Insets are corresponding dark-field scattering and SEM images.

Strong chiroptical effects are also detected from typical near-spherical gold nanocrystals of different shapes placed on gold films. Maximum *g* values from near-cuboctahedral nanoparticle-on-mirror (NPoMs) and nanocube on mirror (NCoMs) can reach 0.5 (Figure 4d–f).

This is much reduced for ultraspherical nanosphere on mirror (USoMs) morphologies, with *g*-values below 0.2 around 650 nm, due to their small reduced morphological defects. Simulations confirm contributions in all cases arise only from imperfect geometries, edge modes, and distinct facets underneath^51^ (Figures S37–S43). Finally, we note that chirality can also come from adapting the geometry of nanocrystals on the gold film: for example, when the lower facet of nanocubes is not parallel to the gold film surface. This is less relevant here due to the ultrasmooth gold mirror and monolayer molecular spacer utilized.

In conclusion, we theoretically and experimentally demonstrate the universal existence of spontaneously formed chirality in nanocrystals synthesized in achiral environments. Using calibrated polarization-dependent scattering, differently shaped gold nanocrystals placed just above a gold film show ultrastrong chiroptical effects in scattering, with maximum *g*-factors reaching 0.9 for NDoMs under symmetrical high-angle illumination. This significant chiroptical effect is found to come from both the chiral nanocrystal geometry and the enhanced effect of edge modes formed underneath. These suppress out-of-plane dipoles while enhancing in-plane dipoles. Coherent superposition of in-plane dipoles leads to strong chiroptical effects in scattering, while coherent superposition of out-of-plane dipoles leads to crescent-shaped scattering patterns. Using unpolarized wide-angle illumination minimizes extrinsic chirality contributions. Our work reveals a wide existence of geometrical and optical chirality in nanocrystals synthesized in achiral environments and provides an effective method to enhance and detect this optical chirality. These findings bring new understanding to nanocrystal chirality and open prospects to applications such as chiral biosensors, chiral anticounterfeiting devices, and chiral nonlinear devices.

## Supporting Information Available

Sample preparation and characterization and electromagnetic simulations, reference scattering spectra and comparison with other works, measured scattering spectra under oblique illuminations, geometrical models of NDs and NDoMs, simulated spectra of NDoMs, influence of different substrates, charge distributions, multidipole decomposition calculations, polarization emission and coherent superposition of dipoles, far-field scattering patterns, simulated results of other NPoMs, and references (PDF)
