# Supplementary material for: Extreme Optical Chirality from Plasmonic Nanocrystals on a Mirror
Source: Nano Lett. 2025 Jan 13;25(3):1158–64. doi: 10.1021/acs.nanolett.4c05668 (PMC11760171; doi:10.1021/acs.nanolett.4c05668)
Supplement: Supplementary file 1 — nl4c05668_si_001.pdf [file nl4c05668_si_001.pdf]

Supporting Information for

Extreme Optical Chirality from Plasmonic Nanocrystals on a Mirror

Yidong Hou[*,#,1,2], Xiu Yang[#,1], Shu Hu[2], Qianqi Lin[2,‡], Jie Zhou[1], Jialong Peng[2,†], Chenyang Guo[2], Shanshan Huang[1], Liangke Ren[1], Ana Sánchez-Iglesias[3,4], Rohit Chikkaraddy[*,2,5] and Jeremy J. Baumberg[*,2]

[1] College of Physical Science and Technology, Sichuan University, China Chengdu 610065, China

[2] NanoPhotonics Centre, Cavendish Laboratory, Department of Physics, University of Cambridge, Cambridge, CB3 0HE, United Kingdom

[3] CIC biomaGUNE, Basque Research and Technology Alliance (BRTA), Donostia-San Sebastián 20014, Spain

[4] Center of Materials Physics, CSIC-UPV, Donostia-San Sebastián 20018, Spain

[5] School of Physics and Astronomy, University of Birmingham, Birmingham B15 2TT, United Kingdom

[#] these authors contributed equally to this work

[*] houyd@scu.edu.cn, r.chikkaraddy@bham.ac.uk, jjb12@cam.ac.uk

Keywords: nano-decahedra, chiroptical effect, nanoparticle on mirror, multiple dipole decomposition theory, photonic spin Hall effect

This supporting information includes:

1. Methods
2. Reference scattering spectra and comparison with other work (Fig. S1 and Table S1)
3. Influence of oblique illumination conditions in experiment (Figs. S2-3)
4. The geometry of NDs and NDoMs (Figs. S4-6)
5. The simulated results of LH and RH NDoMs (Figs. S7-18)
6. The influence of different substrates (Figs. S19-22)
7. Charge distributions (Figs. S23-26)
8. Multidipole decomposition calculations (Figs. S27-29)
9. Polarization emission and coherent superposition of dipoles (Figs. 30-32)
10. Far-field scattering patterns (Figs. 33-35)
11. Simulated results of other NPoMs (Figs. 36-43)
12. References

1. Methods

Sample preparation. Gold decahedra were synthesized using an improved seed growth technique with thermal treatment. The decahedra side length was modified by a regrowth method from gold decahedra of 40 nm in edge length as seeds [41]. The ultra-smooth spherical gold nanoparticles were fabricated by a standard seed growth method. The gold nano-cubes and the spherical gold nanoparticles were purchased from Nanopartz and BBI Solutions, respectively. The Au substrates with rms roughness < 0.2 nm were prepared via a

template stripping method. The 1.3 nm-thick self-assembled monolayer (SAM) of biphenyl-4-thiol (BPT) molecules was prepared on these Au surfaces by immersion in a 1 mM analyte solution for 22 h. Then, the substrates were rinsed with a large amount of ethanol to remove the physically absorbed BPT molecules, and dried with nitrogen gas. The Au nanoparticles were dropcast onto the substrates for 30 seconds before being rinsed with deionized water and dried with nitrogen gas. The short dropcasting time ensures the formation of a low density of Au nanoparticles so they individual single nanoparticles can be distinguished under optical microscopy. Detailed growth, characterisation and modelling is found in [1].

Polarized DF and EM characterization. The polarized DF scattering spectra were collected by a home-built microscope system based on components of an Olympus BX51, as shown by the optical path shown in Figure 1(a). Dark hollow Gaussian beams illuminate the samples from a 100 W halogen white light lamp source through a 100 × DF objective (NA 0.8 Olympus LWD), and the scattering light was collected by the same objective and measured by an Andor Newton EMCCD camera coupled to a Kymera 328i spectrometer. To extract the LCP and RCP components of the scattering light, a broadband quarter wave-plate and a polarization beamsplitter (PBS) were placed before the spectrometer, where this dual optical path after the PBS enabled the simultaneous measurement of LCP and RCP components. The LCP and RCP channels are calibrated by first putting a broad wavelength polarizer in the optical path before the quarter wave-plate, and then calculating the ratio between the two channels from the measured spectra. The total DF scattering patterns were recorded by a charge-coupled device camera (Infinity 2) temporarily inserted before the quarter-waveplate, while the LCP and RCP scattering patterns were recorded by a CMOS camera (Zelux® 1.6 MP Monochrome CMOS Camera) placed temporarily at the position denoted in Figure 1(a). To measure the linearly-polarized scattering patterns, a broadband polarizer was placed temporarily before the quarter-waveplate and the scattering patterns were recorded by the same CMOS camera placed after the splitter. The Stokes images of the DF scattering from each single nanoparticle can then be extracted from these polarized scattering patterns.

The morphology of NPs and NPoMs was characterized by a FEI Helios NanoLab Dual Beam microscope and FEI Tecnai F20 transmission electron microscope, respectively. The size of NPs was determined by TEM image analysis through measuring more than 100 randomly chosen NPs.

Electromagnetic Simulations. The electromagnetic simulations were performed with a finite-difference time-domain (FDTD) method (Lumerical Solutions). To simulate the scattering from NPs on different substrates, perfect matching layers were set in X, Y and Z directions. The dark-hollow Gaussian beam used in experiment was created with customized light sources, where a pupil function was used to filter the spatial frequency of the linearly polarized Gaussian wave. The built-in Gaussian wave in the software can be treated as a series of plane waves with various spatial frequencies (f_x, f_y), i.e. propagating along different directions. The pupil function is given by

$$P(f_x, f_y) = \eta(f_x \text{ or } f_y) * \begin{cases} 1, & 0.75^2 < f_x^2 + f_y^2 < 0.85^2 \\ 0, & \text{otherwise} \end{cases}$$

where $\eta(f_x)$ is a step function. The injection plane of the light source is set to 170 nm

above the focal position where the NP is located. To avoid cancellation of Ez components at the focal point, a half dark-hollow Gaussian beam was employed, and 4 individual simulations were required to complete the simulation of each structure considering the X and Y polarizations and the half cone beams in X and Y directions. The scattering fields and near-fields were recorded by monitors placed in suitable positions. The material parameter of Au and Si was adapted from 'Johnson and Christy', and the refractive indexes of the BPT layer and the glass substrate were set to 1.45 and 1.48. The far-field scattering patterns were extracted with the built-in far-field functions. The circularly-polarized components were obtained through the basis vector transform equations, $(\vec{e}_\theta \pm i\vec{e}_\varphi)/\sqrt{2}$, where $\vec{e}_\theta$ and $\vec{e}_\varphi$ are the basis vectors in the far-field polar coordinates, while the linearly-polarized components were extracted by changing the wave-vector to the Z axis with an idea lens and then calculating the projection of $\vec{E}$ to one direction in the XY plane. The multipole decomposition tool is adapted from the program developed by Tatsuki Hinamoto [2] and transplanted to the platform of Python.

2. The reference scattering spectra and the comparison with other works

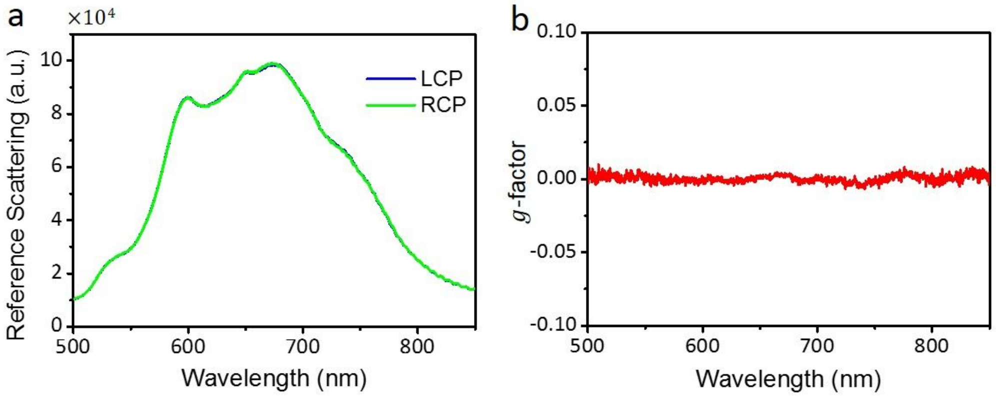

Figure S1. Reference scattering spectra collected by home-built polarization-dependent dark-field scattering spectrometer. (a) LCP (green) and RCP (blue) scattering spectra. (b) Calculated g-factor spectrum. To collect the reference spectra, a standard diffuse reflector was placed at the focus and the scattering light was divided into LCP and RCP components. LCP and RCP spectra are almost the same, leading to extremely small g-factors on the level of 0.01.

Table S1. Comparison of maximum g-factor with other works[3-8]. Red arrows denote illumination directions.

Structure	Illumination Way	Excitation Case	Scattering Case	Maximum g-factor value	Working Wavelength	Ref.
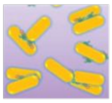	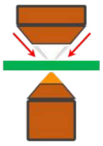	LCP and RCP lights	Total Scattering	**~ 0.2**	~ 600 – 900 nm	[3-4]
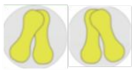	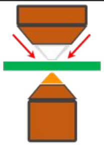	LCP and RCP lights	Total Scattering	**~ 0.28**	~ 650 – 850 nm	[5]
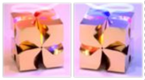	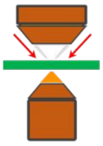	LCP and RCP lights	Total Scattering	**~ 0.8**	~ 600 – 1000 nm	[6]
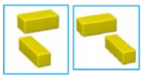	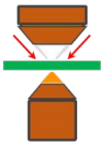	Un-polarized lights	LCP and RCP lights	**~ 1.2**	~ 600 – 1000 nm	[7]
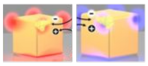	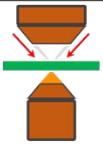	LCP and RCP lights	Total Scattering	**~ 0.1**	~ 600 – 900 nm	[8]
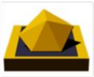	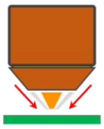	Un-polarized lights	LCP and RCP lights	**~ 0.9**	~ 500 – 850 nm	This Work

3. Influence of oblique illumination conditions in experiment

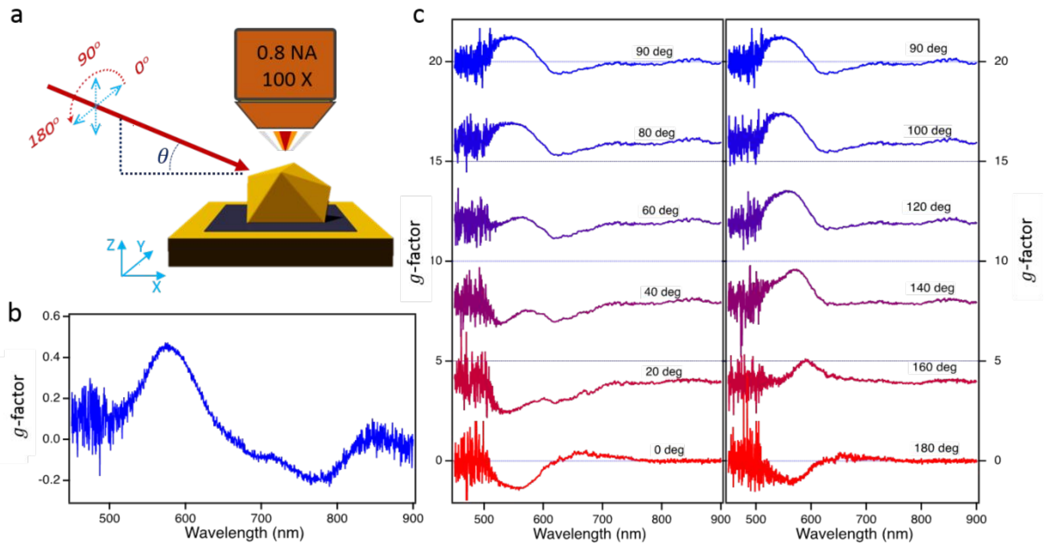

Figure S2. Influence of oblique illumination. (a) Schematic diagram of oblique illumination on NDoM with linearly-polarized lights. Incident light is a supercontinuum laser, and incident angle is ~23°. (b) Measured g-factor spectrum of NDoM under illumination. (c) Measured g-factor spectrum of same NDoM under oblique illumination. Labelled angles denote polarization

direction of incident light. Scattering g-factor depends highly on polarization state of excitation light.

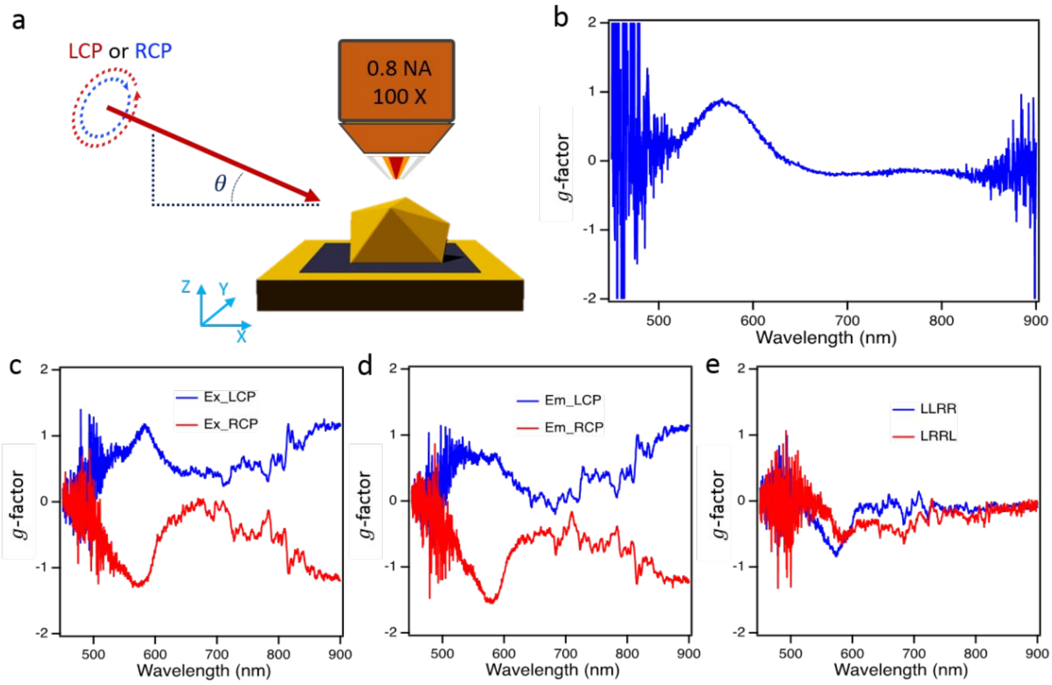

Figure S3. Influence of oblique illumination. (a) Schematic diagram of oblique illumination on NDoM with circularly-polarized light. Incident light is a supercontinuum laser, and incident angle is $\sim 23°$. (b) Measured g-factor spectrum of NDoM under illumination. (c-e) Measured g-factor spectrum of the same NDoM under oblique illumination with circularly-polarized light. Ex_LCP $(= 2 * (I_{LL} - I_{RL})/(I_{LL} + I_{RL}))$ and Ex_RCP $(= 2 * (I_{LR} - I_{RR})/(I_{LR} + I_{RR}))$ denote the scattering g-factors under LCP and RCP excitation respectively. Em_LCP $(= 2 * (I_{LL} - I_{LR})/(I_{LL} + I_{LR}))$ and Em_RCP $(= 2 * (I_{RL} - I_{RR})/(I_{RL} + I_{RR}))$ denote excitation g-factors respectively. LLRR $(= 2 * (I_{LL} - I_{RR})/(I_{LL} + I_{RR}))$ and LRRL $(= 2 * (I_{LR} - I_{RL})/(I_{LR} + I_{RL}))$ denote the g-factor generated in both the excitation and scattering processes. I_{mn} denotes the m-polarized components in scattering under the excitation of n-polarized light.

4. The geometry of NDs and NDoMs

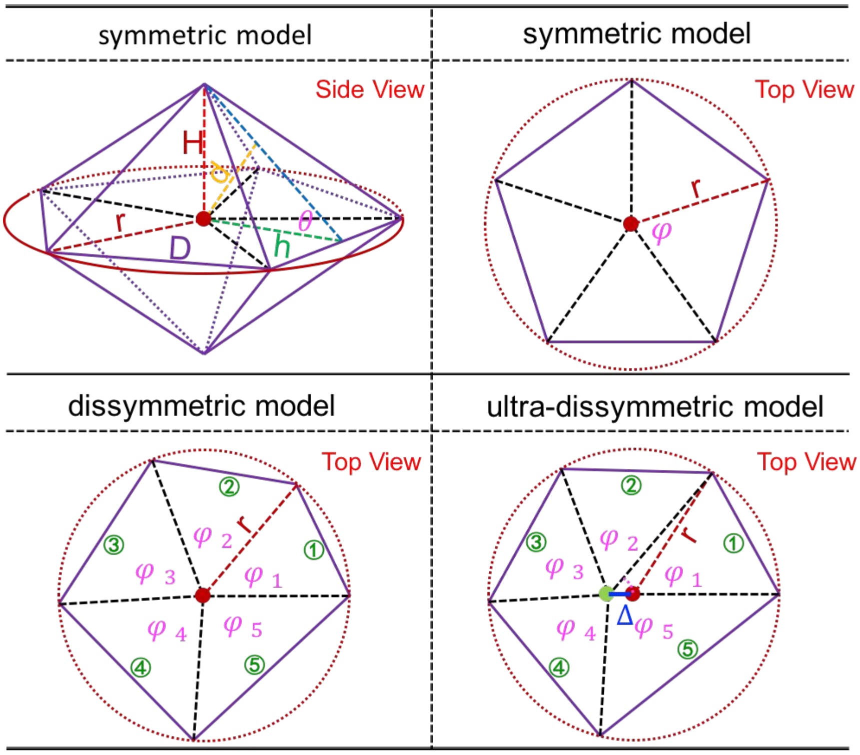

Figure S4. Geometrical parameters of NDs. Side-view and top-view images of an ideal ND are shown on the top-left and top-right, respectively, where all edges of the ideal ND are set to 80 nm in this work (*r* = 80 nm). To generate a chiral ND, we gradually increase the angle with $\varphi_1 = 50°$, $\varphi_2 = 61°$, $\varphi_3 = 72°$, $\varphi_4 = 83°$, and $\varphi_5 = 94°$ (bottom-left image), while keeping the height unchanged (H = 42.06 nm), and the top-view five vertexes inside a circle. To further decrease the symmetry, we move the projection of the remaining two vertices away from the circle center, by a distance $\Delta = 10\ \mathrm{nm}$. All simulation data are based on the ultra-dissymmetric ND model shown in the bottom-right. (It should be noted that this model is a planar chiral structure, and becomes a 3D chiral structure only when there are different heights for the top and bottom pentagonal pyramids. This occurs in the growth process, as has been experimentally demonstrated [9]).

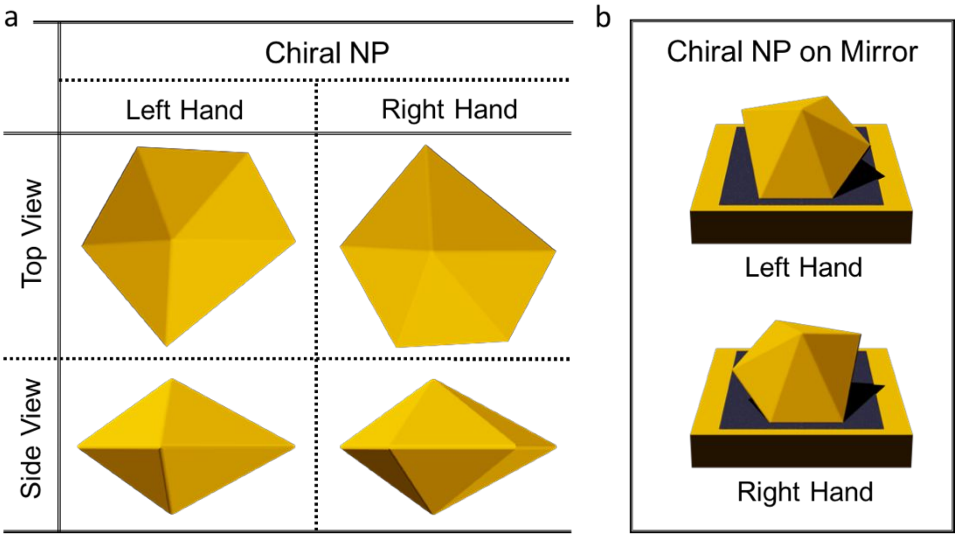

Figure S5. Geometrical models of chiral NDs (a) and chiral NDoM (b). The geometrical parameters of ND are shown in Fig.S4. Increasing direction of angle φ defines left-hand (anti-clockwise) and right-hand (clockwise) particle from the top-view images.

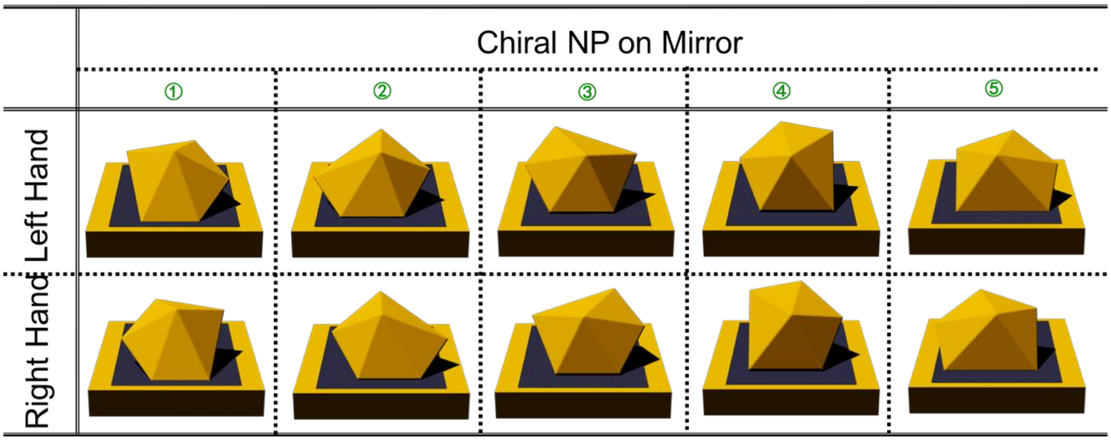

Figure S6. Geometrical models of LH and RH NDoMs. A chiral ND with 10 surfaces can create 10 possible chiral NDoMs with different ND surfaces underneath, where 5 NDoMs are LH and 5 NDoMs are RH. The numbers ① to ⑤ denote the edges shown in Fig.S4.

5. The simulated results of LH and RH NDoMs

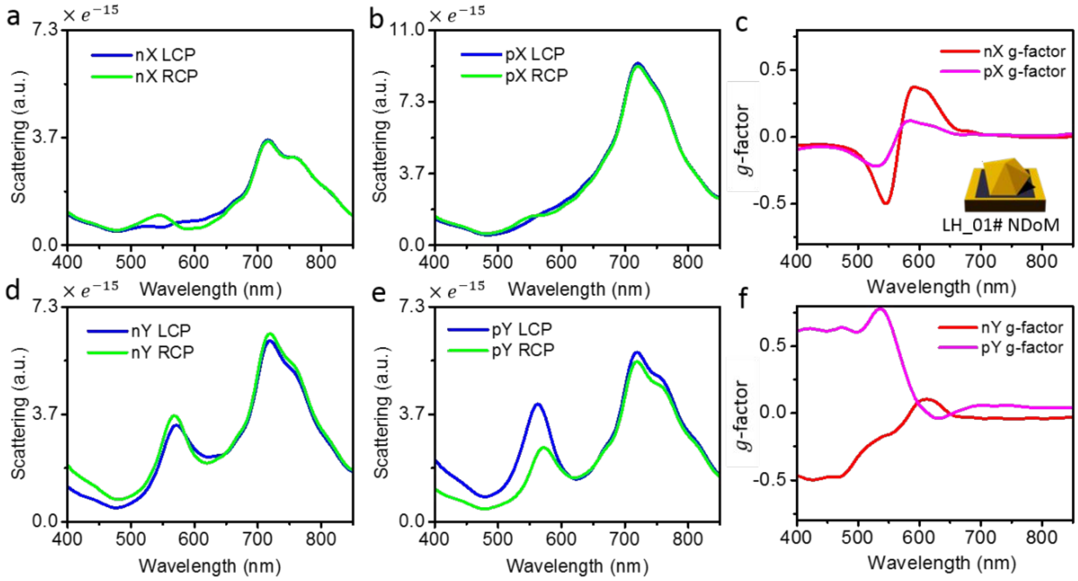

Figure S7. Simulated results of LH_01# NDoM. (a-c) Simulated scattering (a-b) and g-factor (c) spectra of LH_01# NDoM under illumination by X-polarized hollow dark Gaussian beam in the X- (nX) and X+ (pX) half spaces. (d-f) Simulated scattering (d-e) and g-factor (f) spectra of LH_01# NDoM under illumination of Y-polarized hollow dark Gaussian beam in the Y- (nY) and Y+ (pY) half spaces.

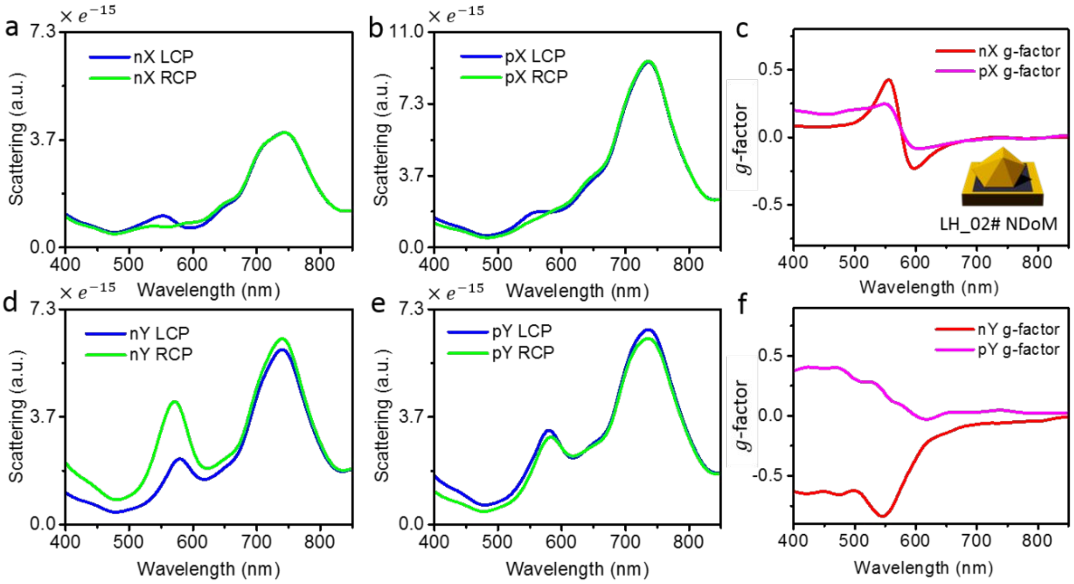

Figure S8. Simulated results of LH_02# NDoM. (a-c) Simulated scattering (a-b) and g-factor (c) spectra of LH_02# NDoM under illumination of X-polarized hollow dark Gaussian beam in the X- (nX) and X+ (pX) half spaces. (d-f) Simulated scattering (d-e) and g-factor (f) spectra of LH_02# NDoM under illumination of Y-polarized hollow dark Gaussian beam in the Y- (nY) and Y+ (pY) half spaces.

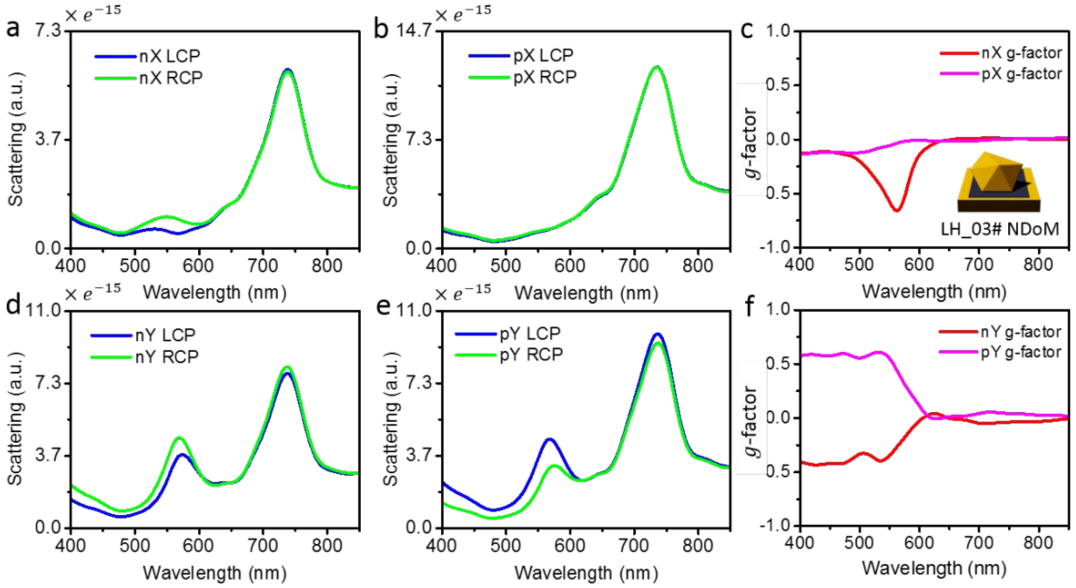

Figure S9. Simulated results of LH_03# NDoM. (a-c) Simulated scattering (a-b) and g-factor (c) spectra of LH_03# NDoM under illumination of X-polarized hollow dark Gaussian beams in the X- (nX) and X+ (pX) half spaces. (d-f) Simulated scattering (d-e) and g-factor (f) spectra of LH_03# NDoM under illumination of Y-polarized hollow dark Gaussian beams in the Y- (nY) and Y+ (pY) half spaces.

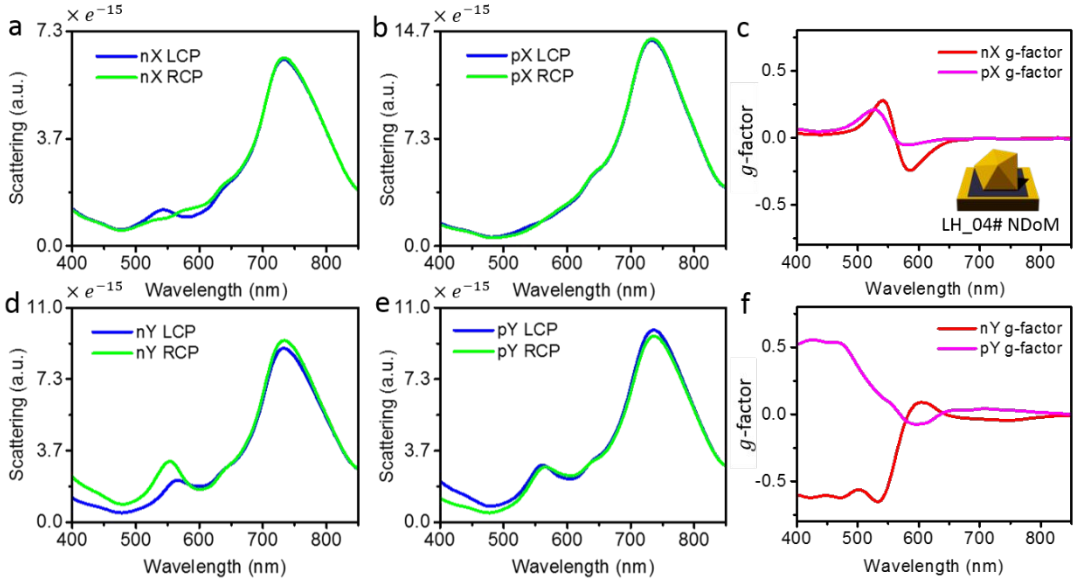

Figure S10. Simulated results of LH_04# NDoM. (a-c) Simulated scattering (a-b) and g-factor (c) spectra of LH_04# NDoM under illumination of X-polarized hollow dark Gaussian beams in the X- (nX) and X+ (pX) half spaces. (d-f) Simulated scattering (d-e) and g-factor (f) spectra of LH_04# NDoM under illumination of Y-polarized hollow dark Gaussian beams in the Y- (nY) and Y+ (pY) half spaces.

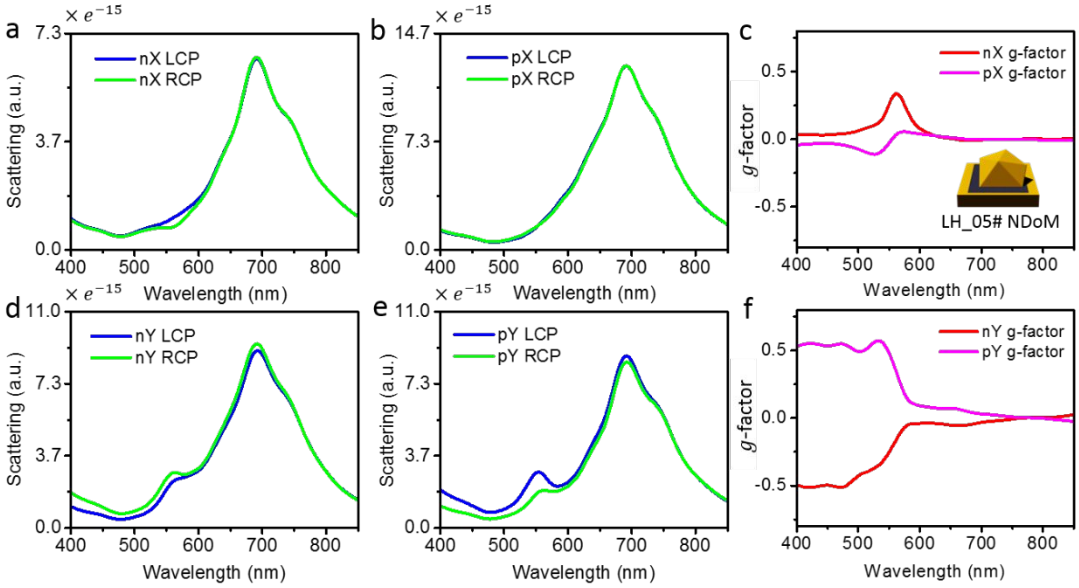

Figure S11. Simulated results of LH_05# NDoM. (a-c) Simulated scattering (a-b) and g-factor (c) spectra of LH_05# NDoM under illumination of X-polarized hollow dark Gaussian beam in the X- (nX) and X+ (pX) half spaces. (d-f) Simulated scattering (d-e) and g-factor (f) spectra of LH_05# NDoM under the illumination of Y-polarized hollow dark Gaussian beam in the Y- (nY) and Y+ (pY) half spaces.

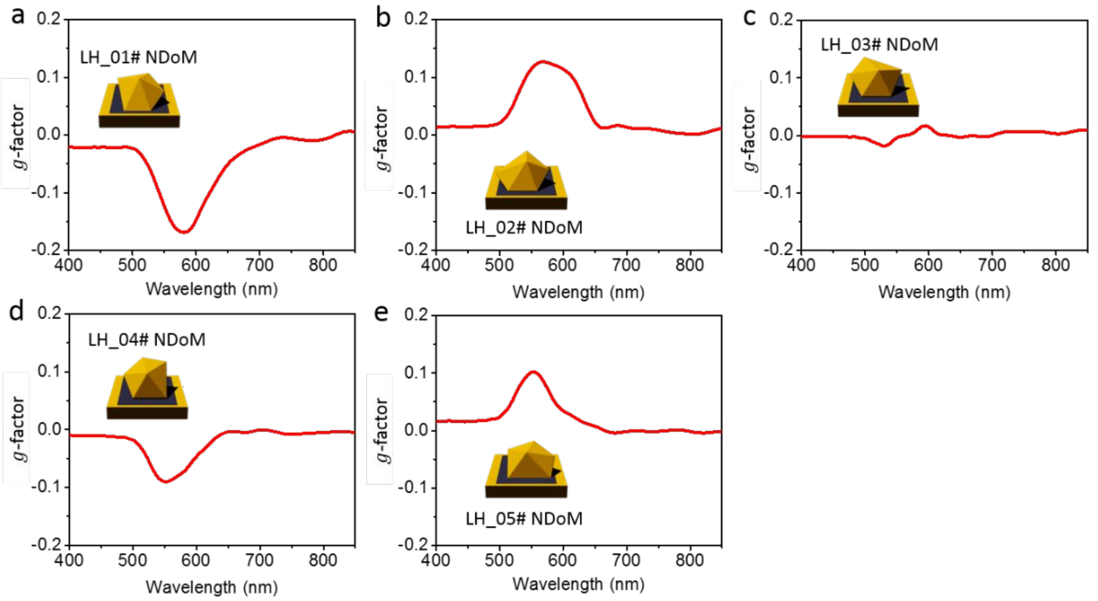

Figure S12. Total g-factor spectra of LH NDoMs from summing the g-factors under illumination by X± and Y± hollow dark Gaussian beams (Figures S7-11).

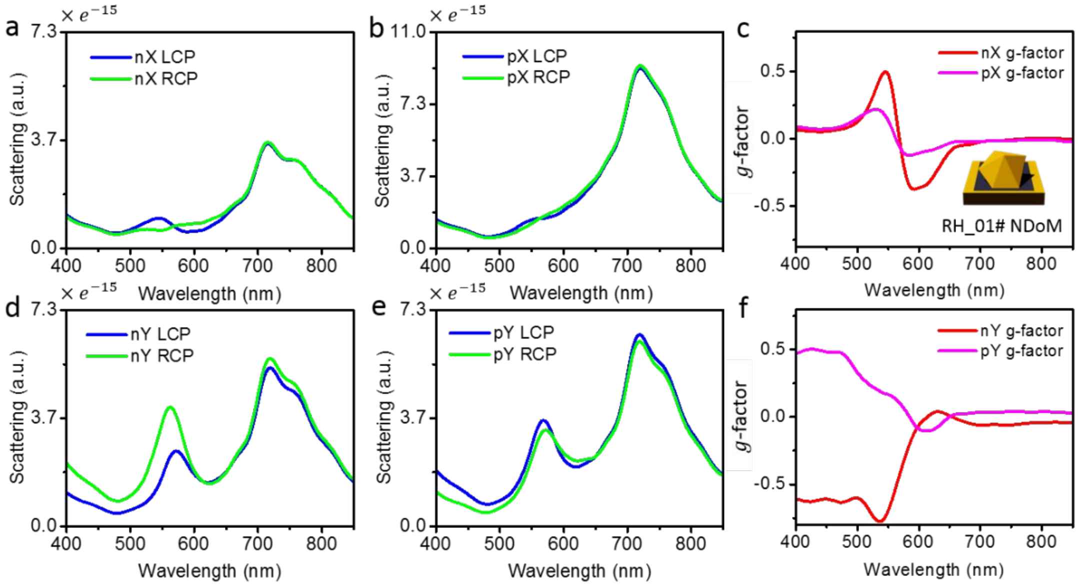

Figure S13. Simulated results of RH_01# NDoM. (a-c) Simulated scattering (a-b) and g-factor (c) spectra of RH_01# NDoM under illumination of X-polarized hollow dark Gaussian beams in the X- (nX) and X+ (pX) half spaces. (d-f) Simulated scattering (d-e) and g-factor (f) spectra of RH_01# NDoM under illumination of Y-polarized hollow dark Gaussian beam in the Y- (nY) and Y+ (pY) half spaces.

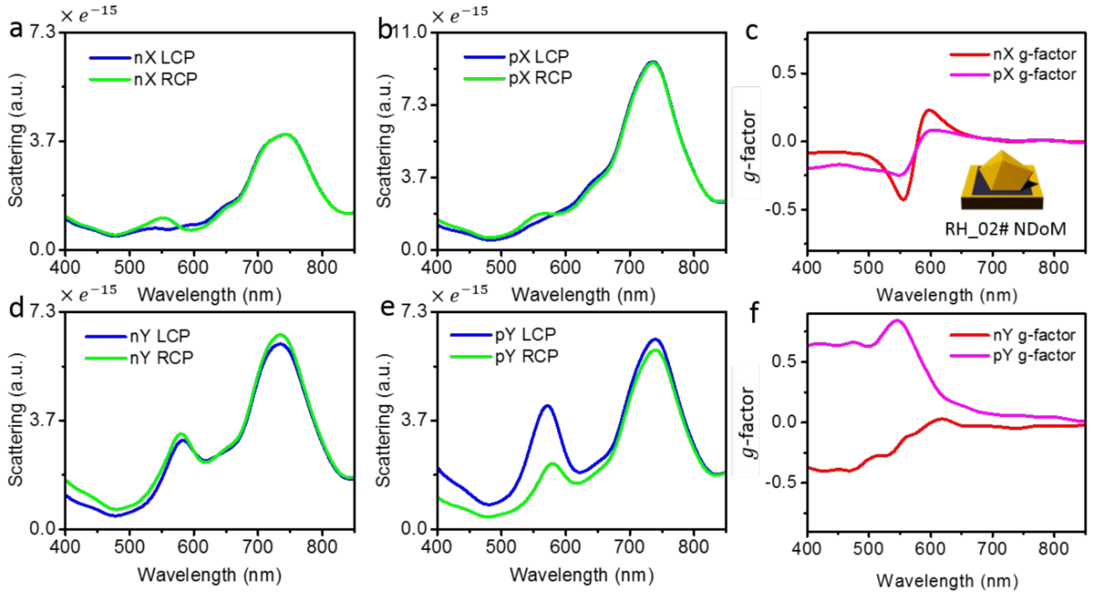

Figure S14. Simulated results of RH_02# NDoM. (a-c) S simulated scattering (a-b) and g-factor (c) spectra of RH_02# NDoM under illumination of X-polarized hollow dark Gaussian beam in the X- (nX) and X+ (pX) half spaces. (d-f) Simulated scattering (d-e) and g-factor (f) spectra of RH_02# NDoM under illumination of Y-polarized hollow dark Gaussian beam in the Y- (nY) and Y+ (pY) half spaces.

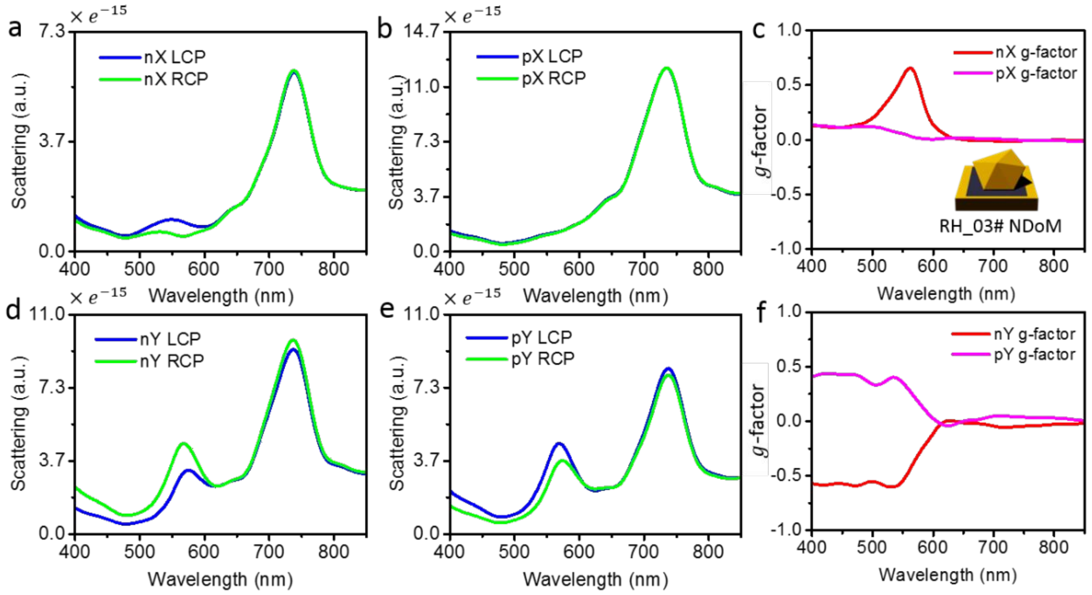

Figure S15. Simulated results of RH_03# NDoM. (a-c) Simulated scattering (a-b) and g-factor (c) spectra of RH_03# NDoM under illumination of X-polarized hollow dark Gaussian beam in the X- (nX) and X+ (pX) half spaces. (d-f) Simulated scattering (d-e) and g-factor (f) spectra of RH_03# NDoM under illumination of Y-polarized hollow dark Gaussian beam in the Y- (nY) and Y+ (pY) half spaces.

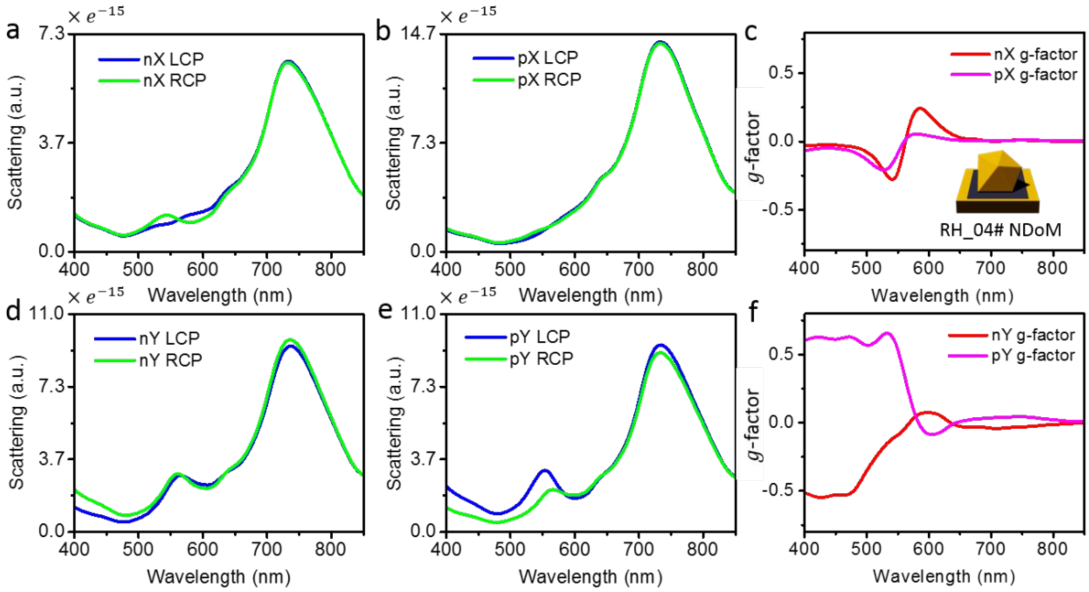

Figure S16. Simulated results of RH_04# NDoM. (a-c) Simulated scattering (a-b) and g-factor (c) spectra of RH_04# NDoM under illumination of X-polarized hollow dark Gaussian beam in the X- (nX) and X+ (pX) half spaces. (d-f) Simulated scattering (d-e) and g-factor (f) spectra of RH_04# NDoM under illumination of Y-polarized hollow dark Gaussian beam in the Y- (nY) and Y+ (pY) half spaces.

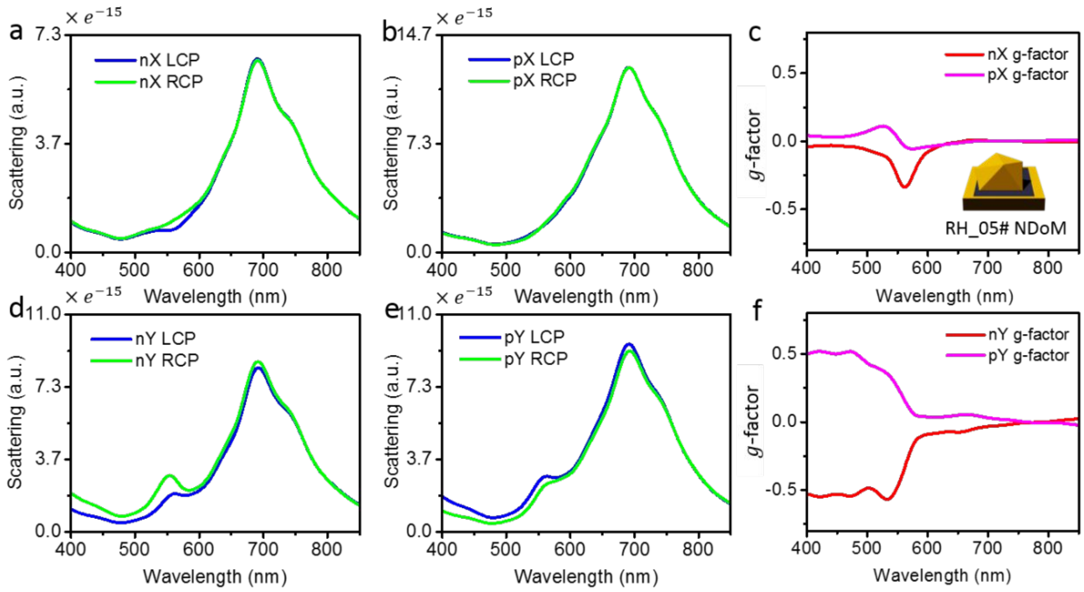

Figure S17. Simulated results of RH_05# NDoM. (a-c) Simulated scattering (a-b) and g-factor (c) spectra of RH_05# NDoM under illumination of X-polarized hollow dark Gaussian beam in the X- (nX) and X+ (pX) half spaces. (d-f) Simulated scattering (d-e) and g-factor (f) spectra of RH_05# NDoM under illumination of Y-polarized hollow dark Gaussian beam in the Y- (nY) and Y+ (pY) half spaces.

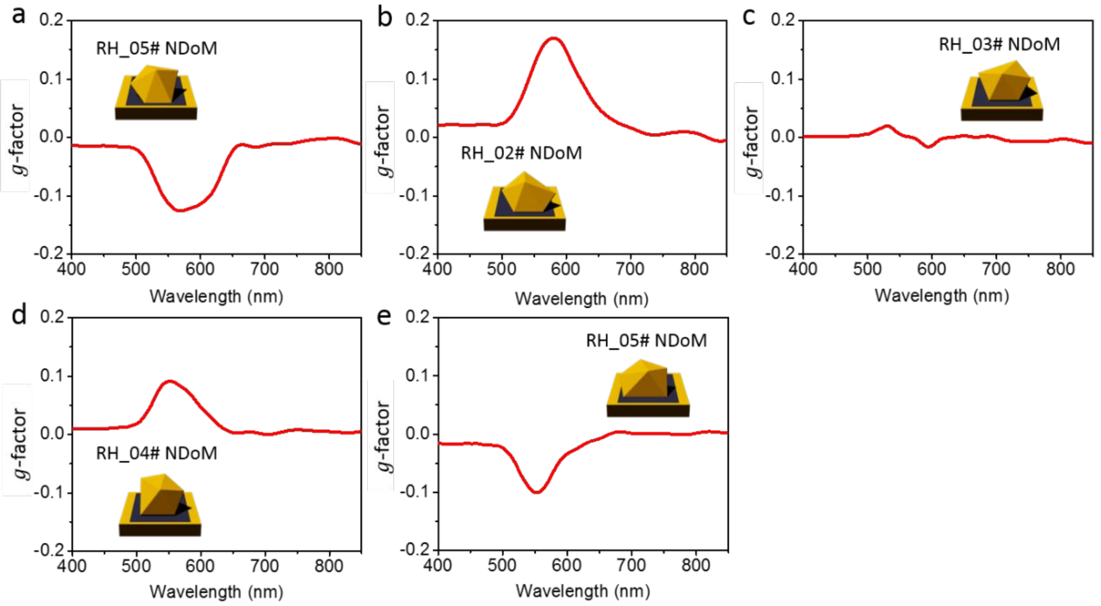

Figure S18. Total g-factor spectra of RH NDoM gtom summing the g-factors under illumination by X± and Y± hollow dark Gaussian beams (Figs. S13-17).

6. The influence of different substrates

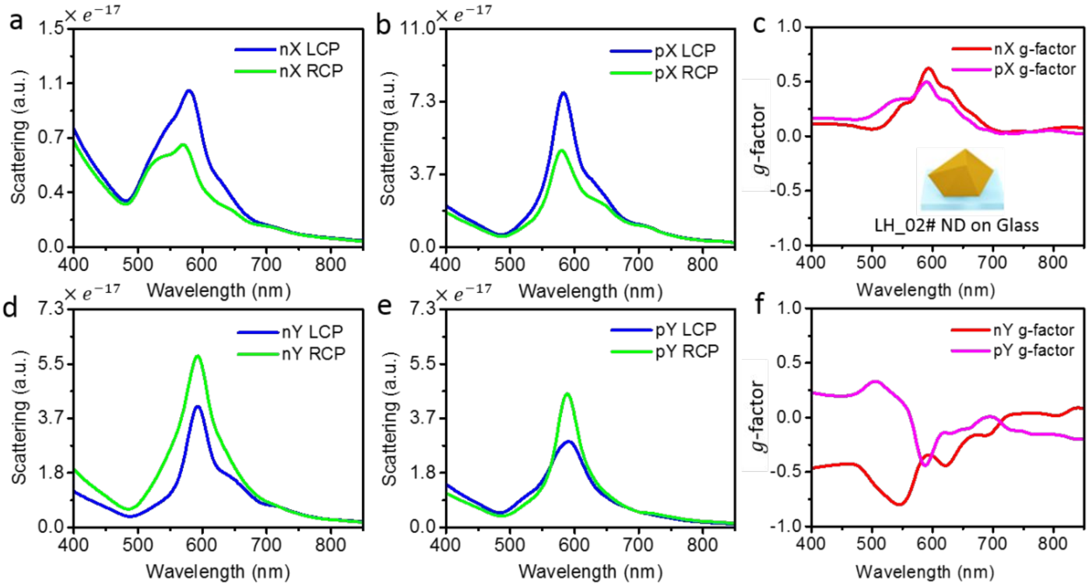

Figure S19. Simulated results of LH_02# ND on glass. (a-c) Simulated scattering (a-b) and g-factor (c) spectra of LH_02# NDoG under illumination of X-polarized hollow dark Gaussian beam in the X- (nX) and X+ (pX) half spaces. (d-f) Simulated scattering (d-e) and g-factor (f) spectra of LH_02# NDoG under illumination of Y-polarized hollow dark Gaussian beam in the Y- (nY) and Y+ (pY) half spaces.

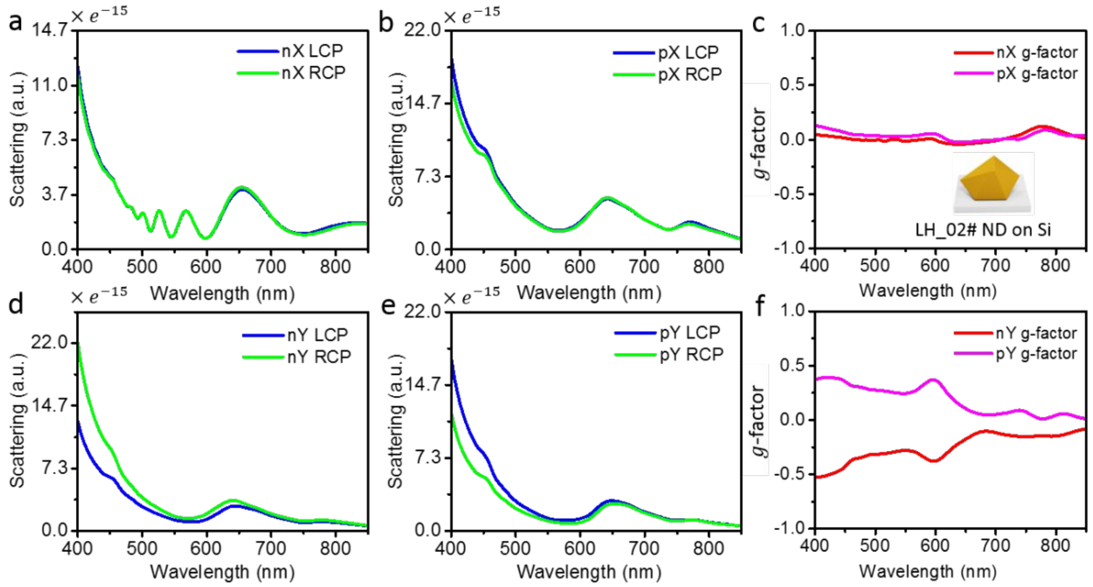

Figure S20. Simulated results of LH_02# ND on Si. (a-c) Simulated scattering (a-b) and g-factor (c) spectra of LH_02# NDoS under illumination of X-polarized hollow dark Gaussian beam in the X- (nX) and X+ (pX) half spaces. (d-f) Simulated scattering (d-e) and g-factor (f) spectra of LH_02# NDoS under illumination of Y-polarized hollow dark Gaussian beam in the Y- (nY) and Y+ (pY) half spaces.

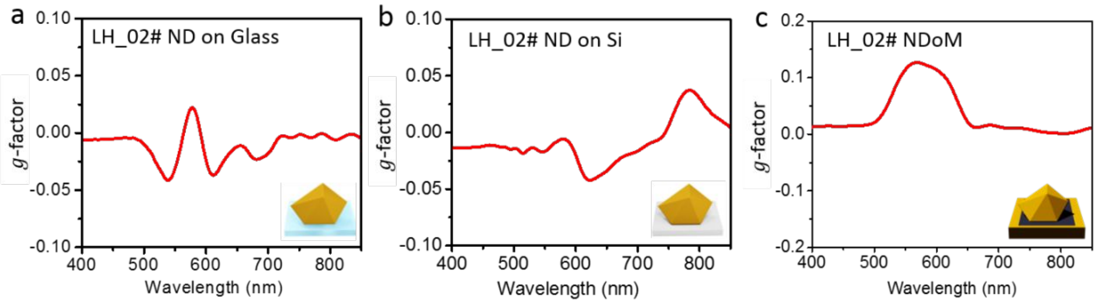

Figure S21. Total g-factor spectra of LH_02# ND on glass (a), Si (b), and gold film (c) by summing the total LCP and RCP light intensities under illuminations by X± and Y± hollow dark Gaussian beams (Figs. S19-20 and Fig. S8).

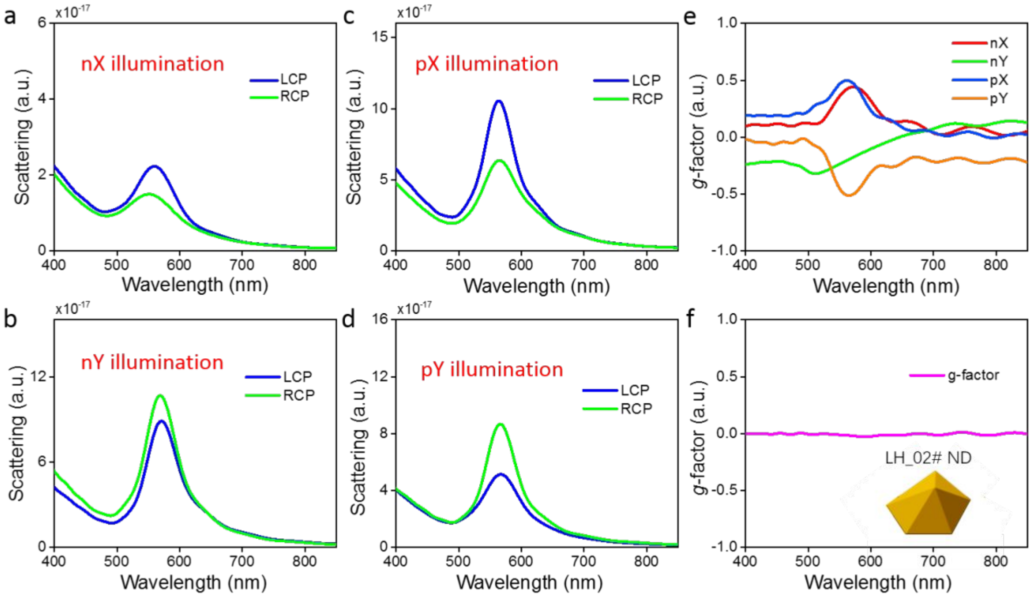

Figure S22. Simulated results from LH_02# ND. (a-d) Simulated scattering (a-d) and g-factor (e-f) spectra of LH_02# ND. (e) Simulated *g*-factor spectra under different illumination conditions. (f) Total *g*-factor spectra extracted by summing the LCP and RCP light intensities under illumination by X± and Y± hollow dark Gaussian beams. nX and pX denote the illumination of X-polarized hollow dark Gaussian beam in the X- (nX) and X+ (pX) half spaces. nY and pY denote the illumination of Y-polarized hollow dark Gaussian beam in the Y- (nY) and Y+ (pY) half spaces.

7. The charge distributions

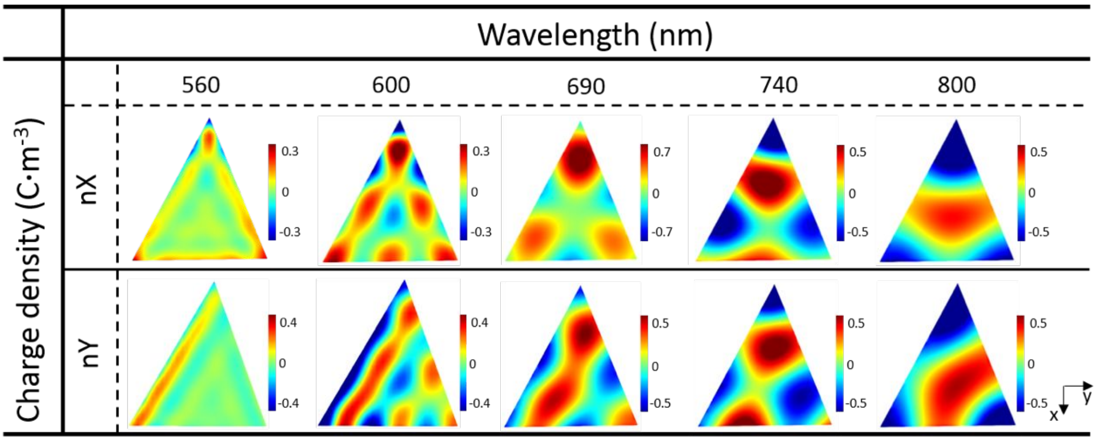

Figure S23. Simulated charge distributions on the underside of the ND using the structure of LH_02# NDoM.

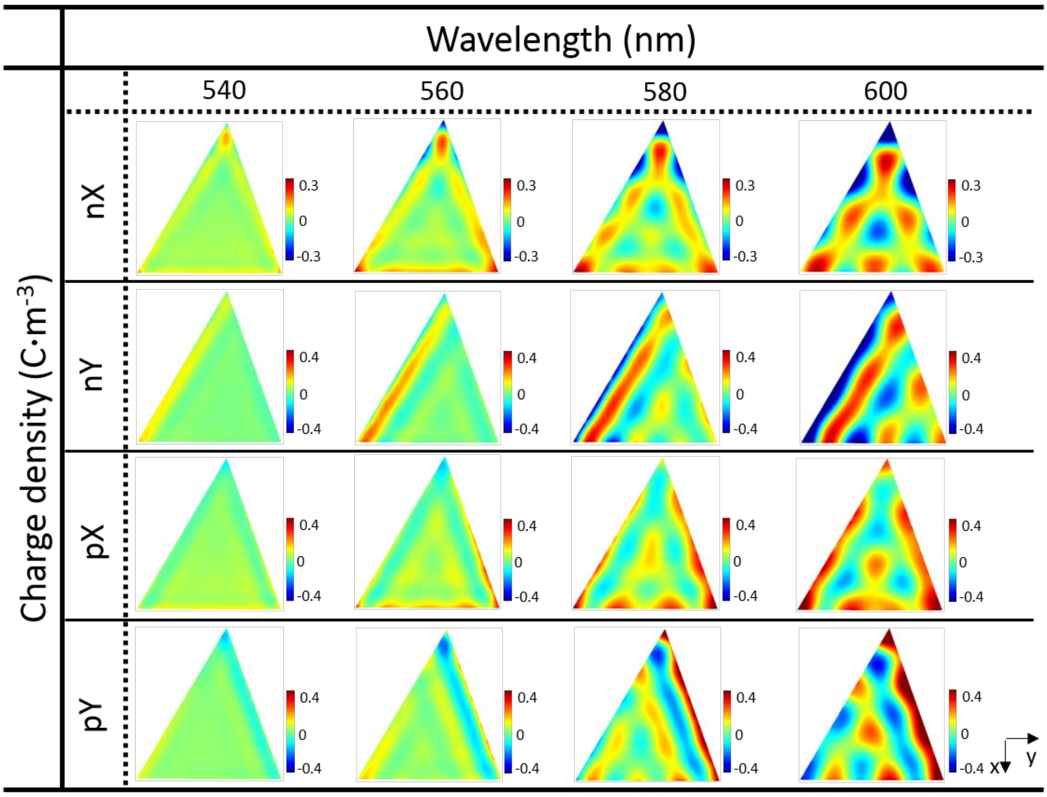

Figure S24. Simulated charge distribution on the underside of the ND using the structure of LH_02# NDoM.

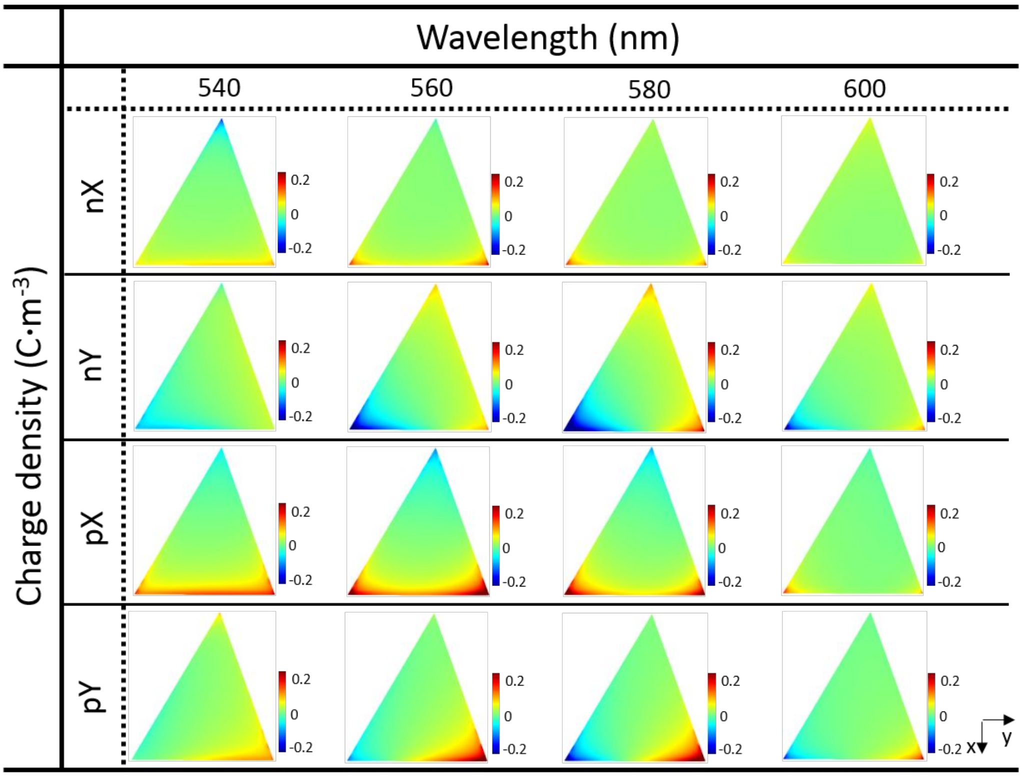

Figure S25. Simulated charge distribution on the underside of the ND using the structure of LH_02# ND on glass.

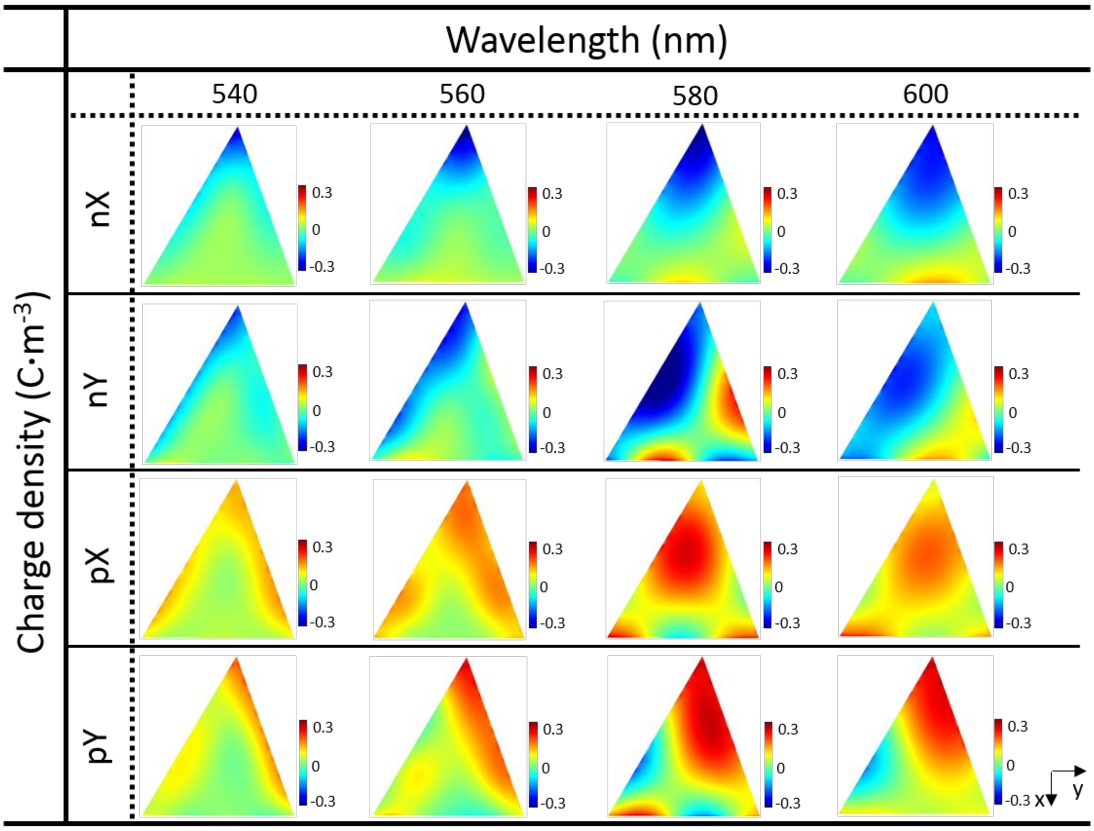

Figure S26. Simulated charge distribution on the underside of the ND using the structure of LH_02# ND on silicon.

8. The multidipole decomposition calculations

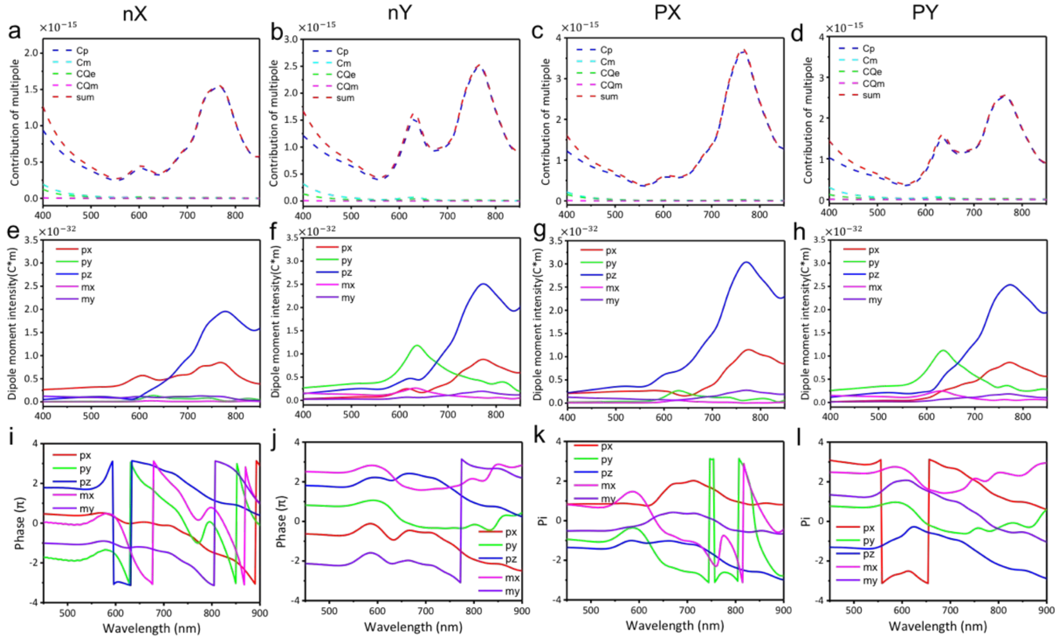

Figure S27. Calculated equivalent current dipoles from LH_02# NDoM under illumination by X- and Y-polarized hollow dark Gaussian beams in the X- (nX), X+ (pX), Y- (nY), and Y+ (pY) half spaces. (a-d) Contribution of each dipole to the total scattering intensity. (e-h) Intensity of each equivalent dipole. (i-l) Phase of each equivalent dipole. Px, Py and Pz denote the electric dipole

components along the X, Y and Z axes. Mx and My denote the magnetic dipole components along the X and Y axes.

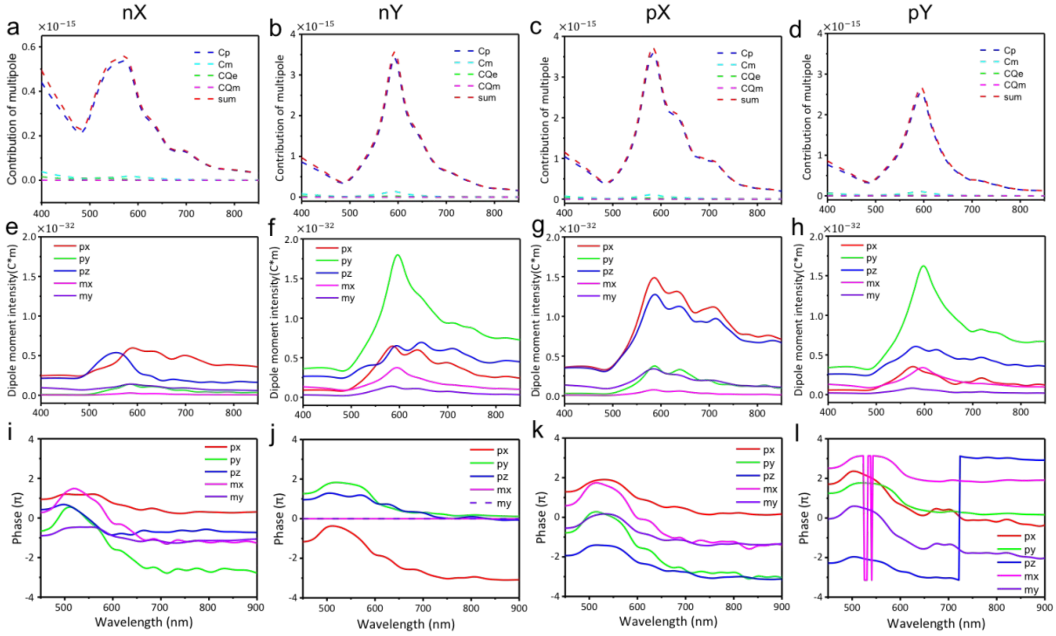

Figure S28. Calculated equivalent current dipoles from LH_02# ND on glass under illumination by X- and Y-polarized hollow dark Gaussian beams in the X- (nX), X+ (pX), Y- (nY), and Y+ (pY) half spaces. (a-d) Contribution of each dipole to the total scattering intensity. (e-h) Intensity of each equivalent dipole. (i-l) Phase of each equivalent dipole. Px, Py and Pz denote the electric dipole components along the X, Y and Z axes. Mx and My denote the magnetic dipole components along the X and Y axes.

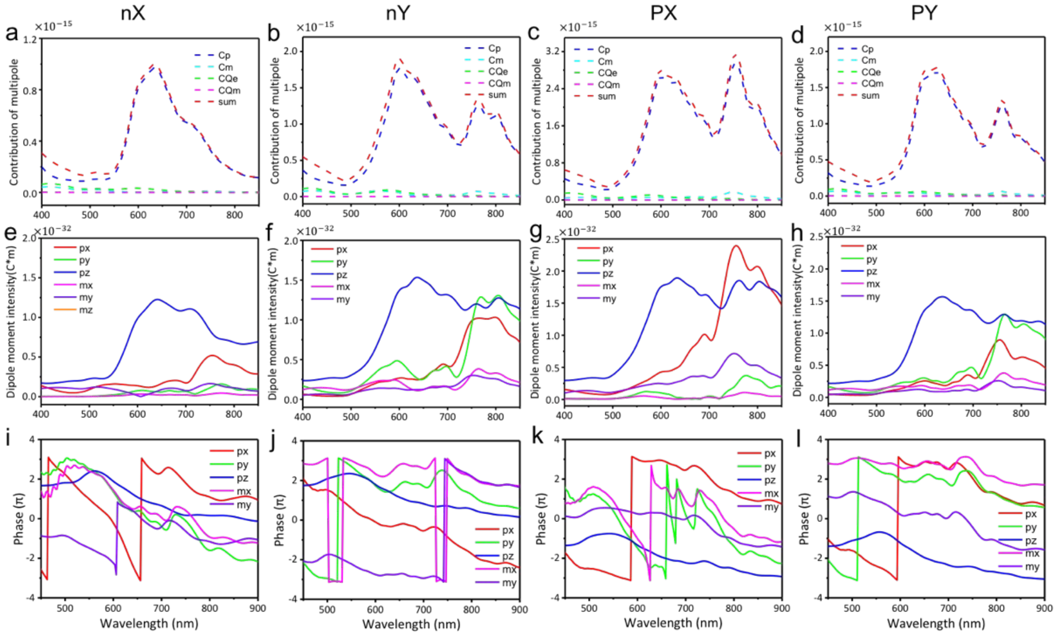

Figure S29. Calculated equivalent current dipoles from LH_02# ND on Si under illumination by X- and Y-polarized hollow dark Gaussian beams in the X- (nX), X+ (pX), Y- (nY), and Y+ (pY) half

spaces. (a-d) Contribution of each dipole to the total scattering intensity. (e-h) Intensity of each equivalent dipole. (i-l) Phase of each equivalent dipole. Px, Py and Pz denote the electric dipole components along the X, Y and Z axes. Mx and My denote the magnetic dipole components along the X and Y axes.

9. Polarization emission and coherent superposition of dipoles

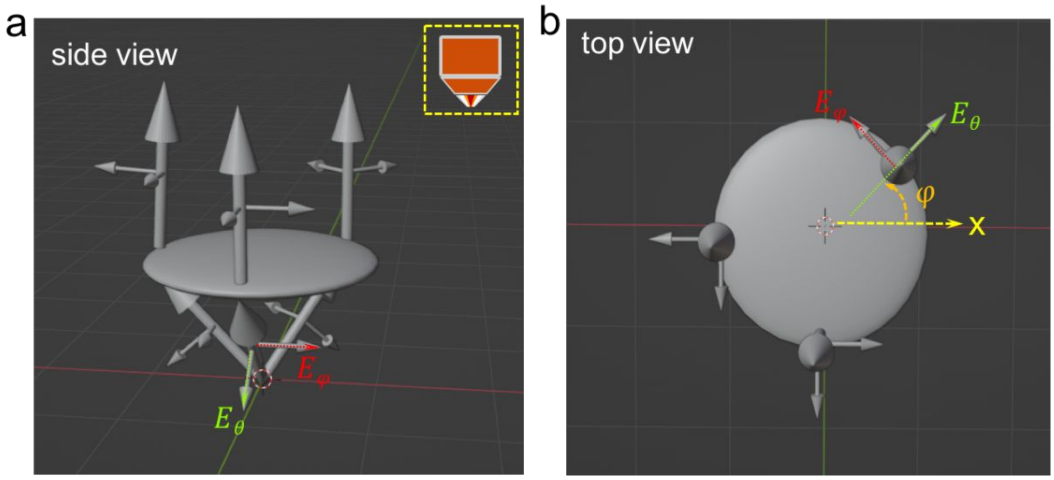

Figure S30. Schematic diagram of the polarization state of scattered light in the far-field. (a) Side view schematic diagram of the scattered light collecting by an ideal lens in the far-field. The polarization state of the scattered light before the lens is denoted by E_θ and E_φ. (b) Side view schematic of the scattered light after the lens in the far-field. As in experiments, all of the electric fields $E_{\theta,\varphi}$ are converted to electric fields in a plane (XY plane), and then $E_{X,Y}$ can be easily extracted using $E_X = E_\theta \cos\varphi - E_\varphi \sin\varphi$ and $E_Y = E_\varphi \cos\varphi + E_\theta \sin\varphi$.

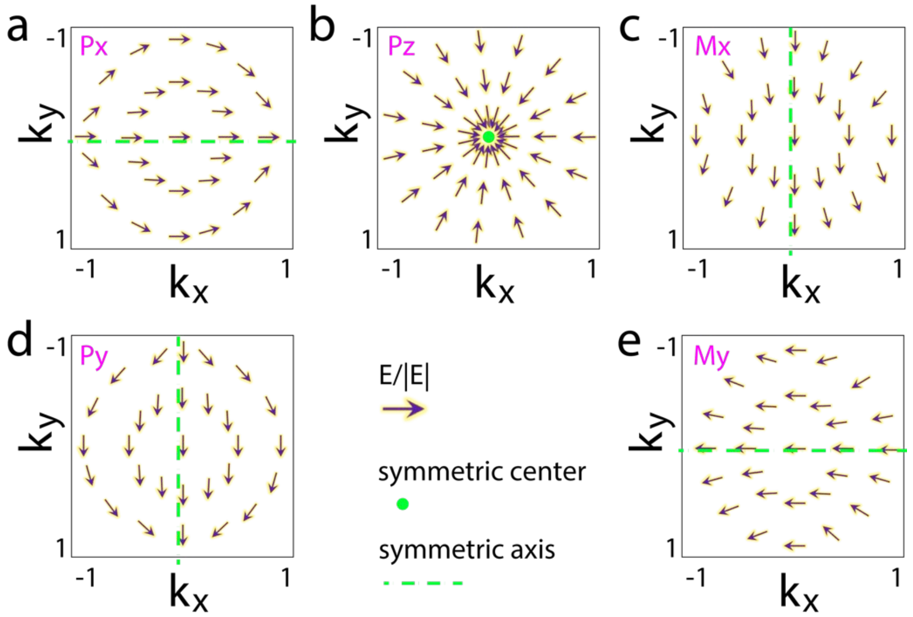

Figure S31. Calculated polarization state of scattered light from ideal dipoles collected by an ideal lens (as in Fig. S30). Green dashed lines denote the symmetry axis of the polarization distributions of Px, Py, Mx and My, and the green point denotes the symmetric center of Pz.

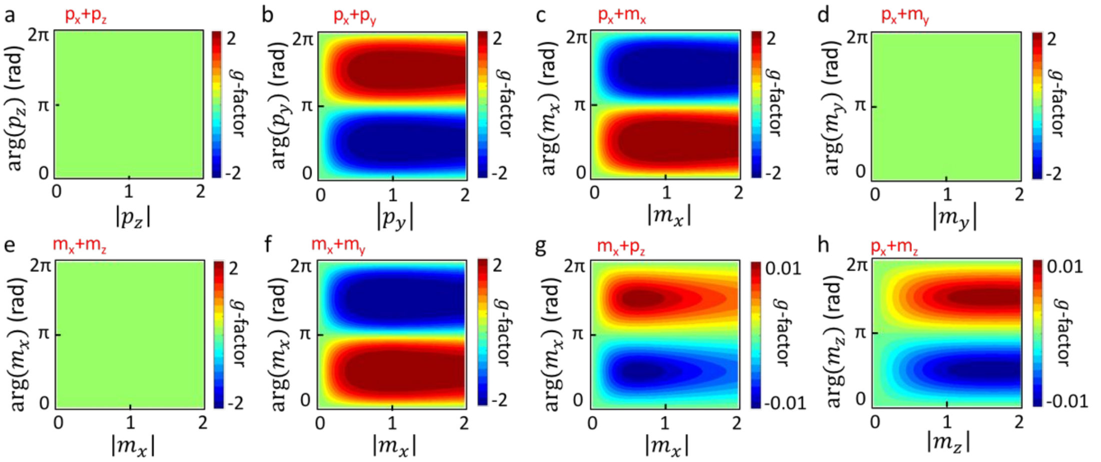

Figure S32. Scattering g-factor in the space of Z+ from the coherent superposition of dipoles. (a) $p_x + p_z$; (b) $p_x + p_y$; (c) $p_x + m_x$; (d) $p_x + m_y$; (e) $m_x + m_z$; (f) $m_x + m_y$; (g) $m_x + p_z$; (h) $p_x + m_z$. The superposition of dipoles which have the same symmetry axis in the polarization distributions of scattered light will not generate strong chiroptical effects, such as Px + Pz, Px + My, Mx + Mz, Mx + Pz, and Px + Mz.

10. Far-field scattering patterns

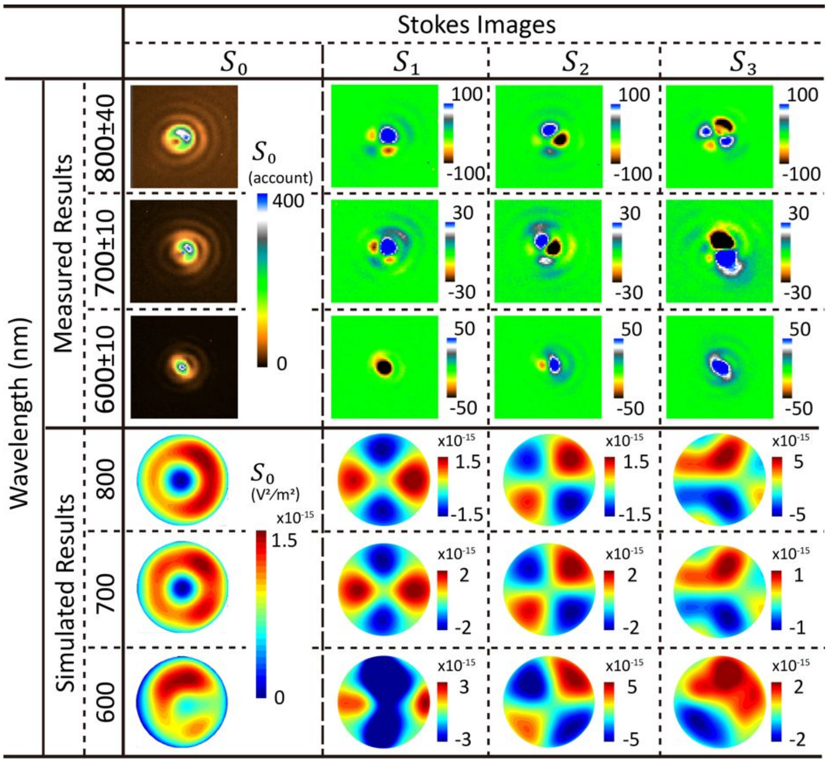

Figure S33. Measured and simulated Stokes images of far-field scattering at different spectral wavelengths from an NDoM. Simulated NDoM model is LH #2, and simulated Stokes images sum those generated by half hollow Gaussian beams in X and Y polarizations.

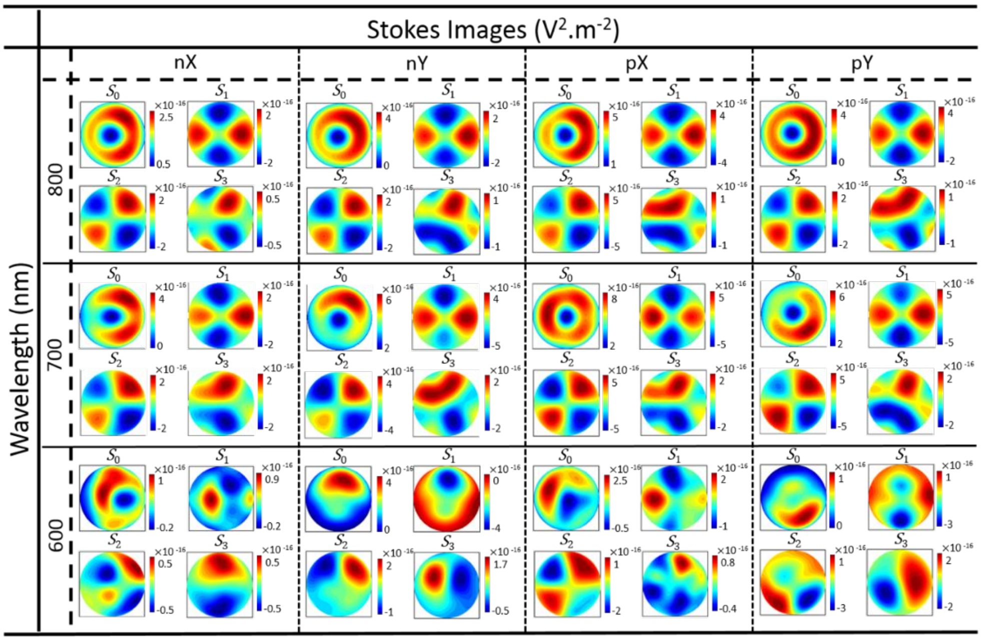

Figure S34. Calculated far-field scattering Stokes images from the LH_02# NDoM collected by an ideal lens. nX and pX denote illumination of X-polarized hollow dark Gaussian beams in the X- (nX) and X+ (pX) half spaces. nY and pY denote illumination of Y-polarized hollow dark Gaussian beams in the Y- (nY) and Y+ (pY) half spaces.

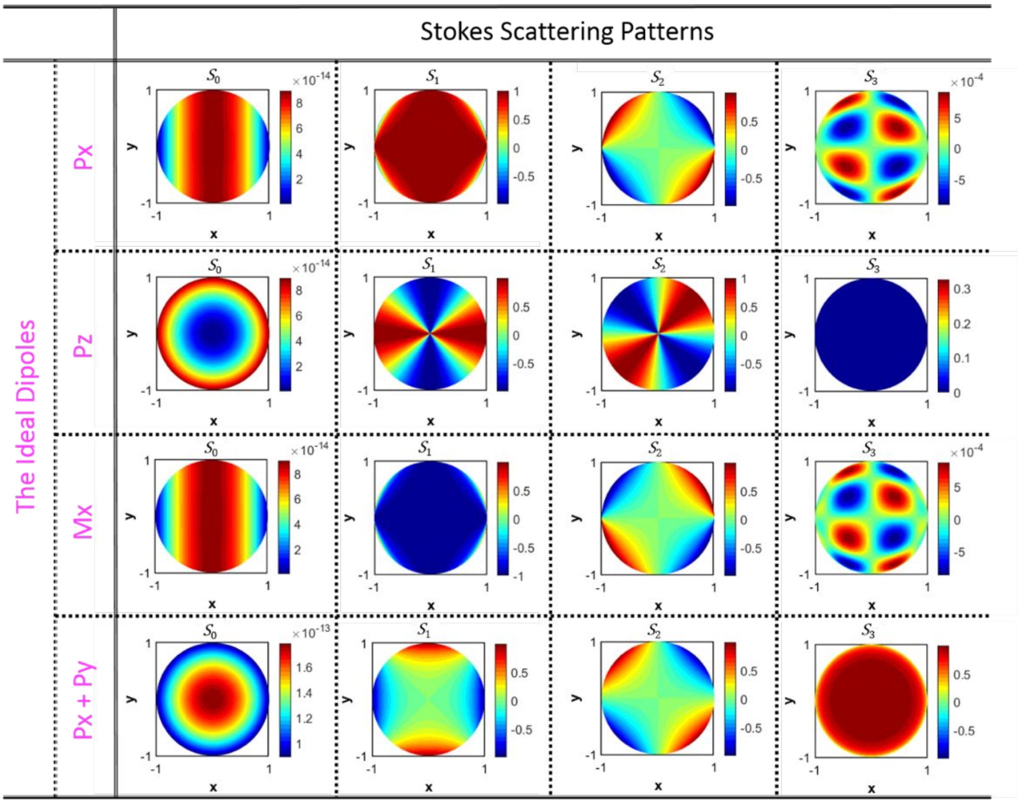

Figure S35. Calculated far-field scattering Stokes images from the ideal electric dipoles (Px, Pz, and Px + Pz).

11. Simulated results of other NPoMs

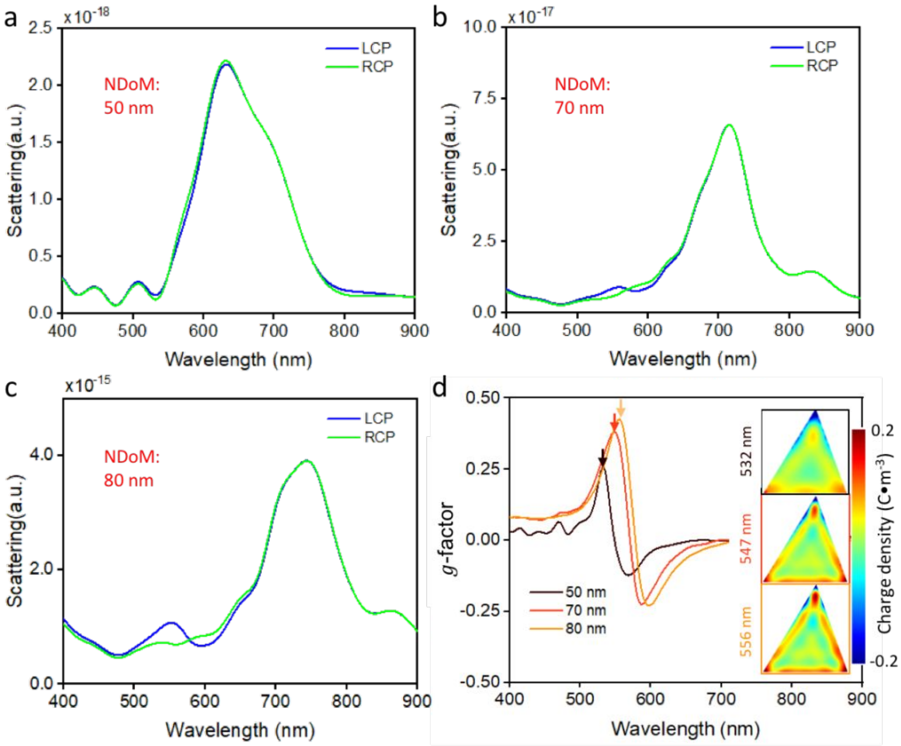

Figure S36. Simulated scattering (a-c) and *g*-factor (d) spectra from LH #2 NDoMs with facets of (a) 50 nm, (b) 70 nm, and (c) 80 nm. Inset images show charge distributions at the chiroptical resonance peaks. Illumination condition is X-polarized hollow dark Gaussian beam in the X- half space (nX).

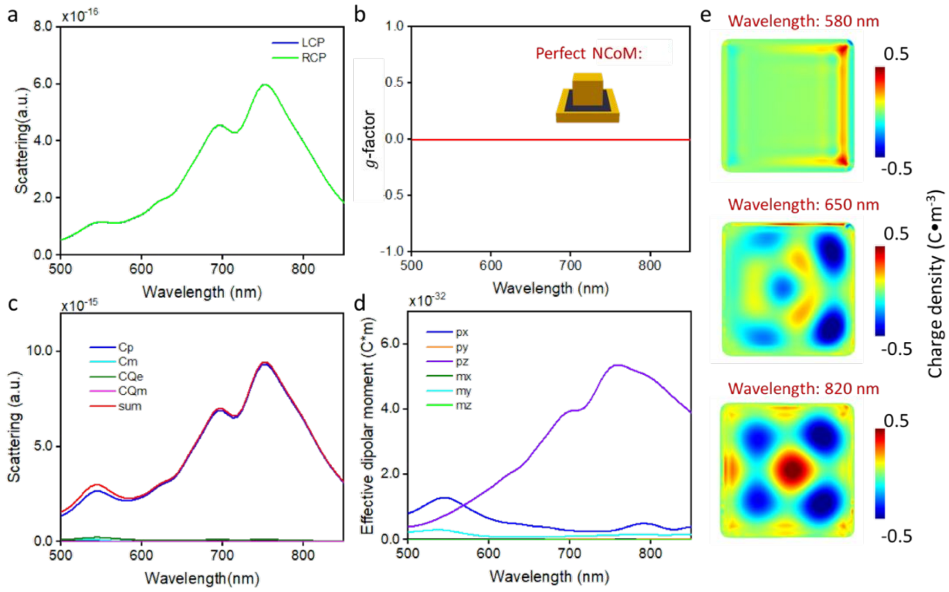

Figure S37. (a,b) Simulated scattering (a) and *g*-factor (b) spectra from a perfect NCoM. (c-d) Calculated equivalent current dipole intensities (c) and their contributions to the total scattering intensity (d). px, py and pz denote the electric dipole components along the X, Y and Z axes, mx, my and mz denote the corresponding magnetic dipole components. (e) Charge distributions on the underside facet of a perfect NC. Illumination condition is X-polarized hollow dark Gaussian beams in

the X+ half space (pX).

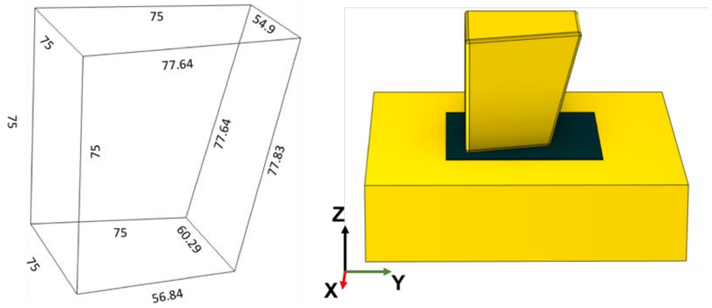

Figure S38. Geometrical model of chiral NC (left) and corresponding NCoM (right) with two tilted facets. Labels denote side lengths in nanometers.

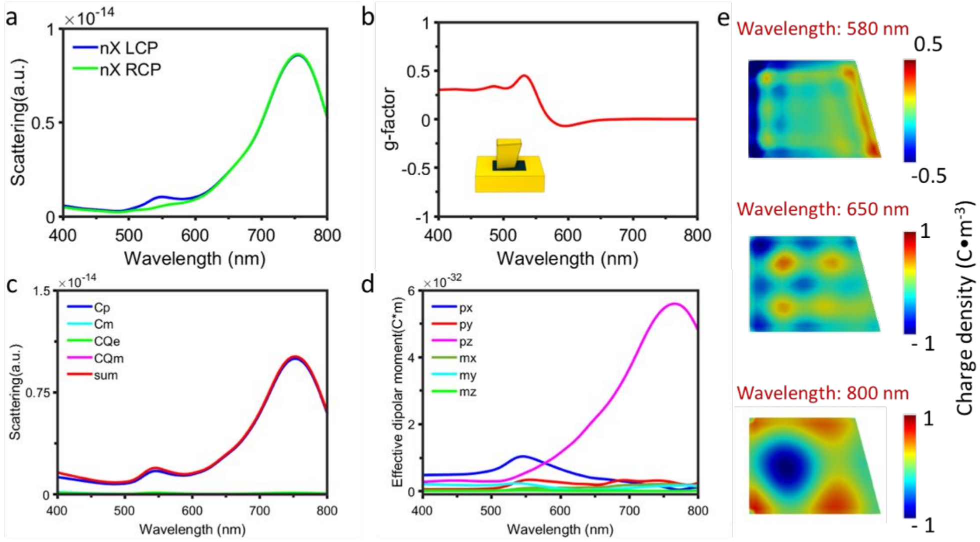

Figure S39. (a,b) Simulated scattering (a) and *g*-factor (b) spectra from an imperfect NCoM with two tilted facets. (c,d) Calculated equivalent current dipole intensities (c) and their contributions to the total scattering intensity (d). (e) Charge distributions on the underside facets of an imperfect NC. Dipoles and illumination as above.

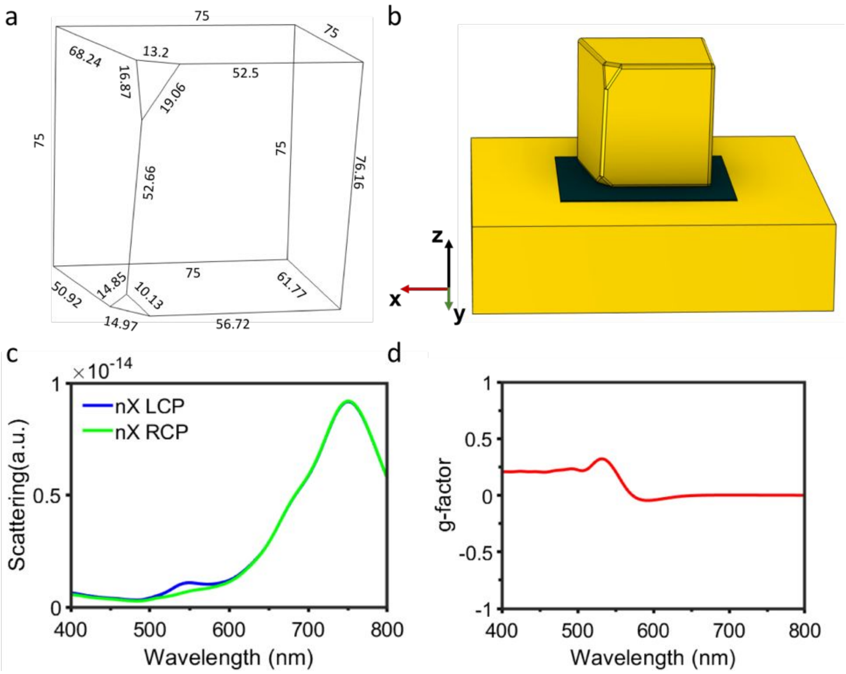

Figure S40. (a,b) Geometrical model of chiral NC (a) and NCoM (b) with two tilted facets and two truncated corners. Numbers denote the side lengths in nanometers. (c,d) Simulated scattering (c) and *g*-factor (d) spectra. Illumination condition is X-polarized hollow dark Gaussian beams in the X- half space (nX).

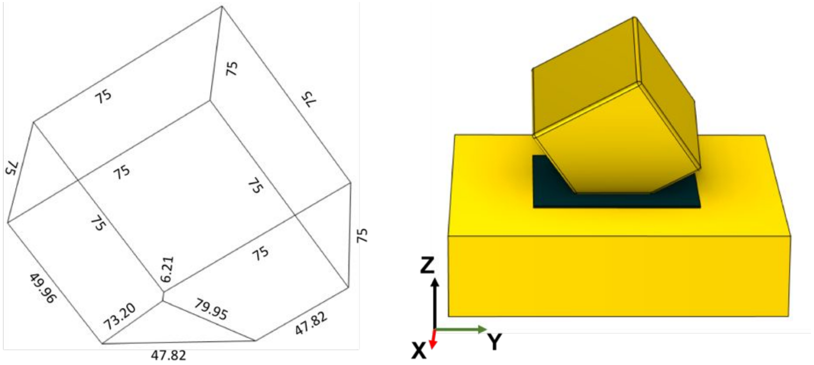

Figure S41. Geometrical model of chiral NC (left) and NCoM (right) with one heavily truncated corner. Numbers denote side lengths in nanometers.

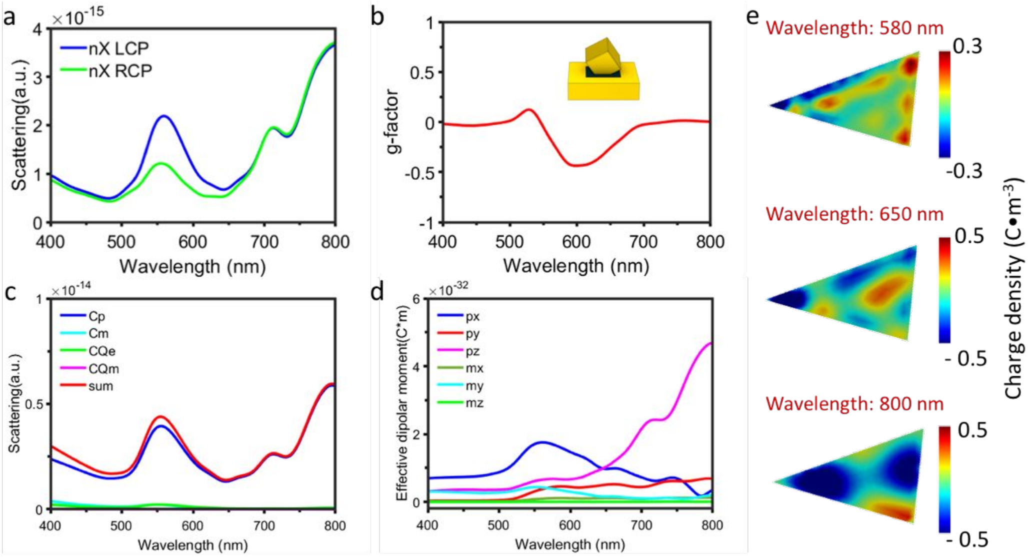

Figure S42. (a,b) Simulated scattering (a) and *g*-factor (b) spectra from an imperfect NCoM with one heavily truncated corner. (c,d) Calculated equivalent current dipole intensities (c) and their contributions to the total scattering intensity (d). (e) Charge distributions on the underside facet of imperfect NCs. Dipoles and illumination as above.

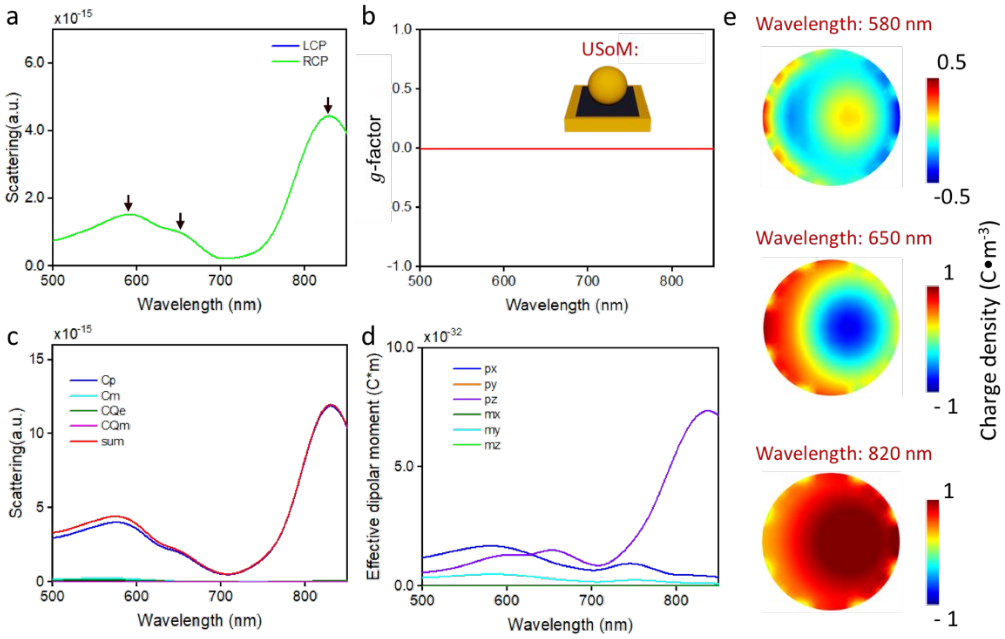

Figure S43. (a,b) Simulated scattering (a) and *g*-factor (b) spectra from a USoM. (c,d) Calculated equivalent current dipole intensities (c) and their contributions to the total scattering intensity (d). Dipoles and illumination as above.

12. References:

[1] S. Hu *et al.*, Full Control of Plasmonic Nanocavities Using Gold Decahedra-on-Mirror Constructs with Monodisperse Facets, Adv. Sci., 2207178 (2023).

[2] T. Hinamoto and M. Fujii, MENP: an open-source MATLAB implementation of multipole expansion for nanophotonics, Osa Continuum **4**, 1640 (2021).

[3] L.-Y. Wang, K. W. Smith, S. Dominguez-Medina, N. Moody, J. M. Olson, H. Zhang, W.-S. Chang, N. Kotov, and S. Link, Circular differential scattering of single chiral self-assembled gold nanorod dimers, Acs Photonics **2**, 1602 (2015).

[4] Q. Zhang *et al.*, Unraveling the origin of chirality from plasmonic nanoparticle-protein complexes, Science **365**, 1475 (2019).

[5] K. W. Smith *et al.*, Chiral and achiral nanodumbbell dimers: the effect of geometry on plasmonic properties, ACS nano **10**, 6180 (2016).

[6] J. Karst, N. H. Cho, H. Kim, H.-E. Lee, K. T. Nam, H. Giessen, and M. Hentschel, Chiral scatterometry on chemically synthesized single plasmonic nanoparticles, ACS Nano **13**, 8659 (2019).

[7] S. Zhou, J. Bian, P. Chen, M. Xie, J. Chao, W. Hu, Y. Lu, and W. Zhang, Polarization-dispersive imaging spectrometer for scattering circular dichroism spectroscopy of single chiral nanostructures, Light: Science & Applications **11**, 64 (2022).

[8] S. Lee *et al.*, Unraveling the Chirality Transfer from Circularly Polarized Light to Single Plasmonic Nanoparticles, Angewandte Chemie **136**, e202319920 (2024).

[9] B. Goris *et al.*, Measuring lattice strain in three dimensions through electron microscopy, Nano letters **15**, 6996 (2015).
